# How a Fully Automated eHealth Program Simulates Three Therapeutic Processes: A Case Study

**DOI:** 10.2196/jmir.5415

**Published:** 2016-06-28

**Authors:** Marianne T. S Holter, Ayna Johansen, Håvar Brendryen

**Affiliations:** ^1^The Norwegian Centre for Addiction ResearchInstitute of Clinical Medicine, Faculty of MedicineUniversity of OsloOsloNorway; ^2^Centre for the Study of Mind in NatureFaculty of HumanitiesUniversity of OsloOsloNorway

**Keywords:** Internet, eHealth, telemedicine, behavior therapy, motivational interviewing, working alliance, intervention mapping, smoking cessation, cell phones, text messaging

## Abstract

**Background:**

eHealth programs may be better understood by breaking down the components of one particular program and discussing its potential for interactivity and tailoring in regard to concepts from face-to-face counseling. In the search for the efficacious elements within eHealth programs, it is important to understand how a program using lapse management may simultaneously support working alliance, internalization of motivation, and behavior maintenance. These processes have been applied to fully automated eHealth programs individually. However, given their significance in face-to-face counseling, it may be important to simulate the processes simultaneously in interactive, tailored programs.

**Objective:**

We propose a theoretical model for how fully automated behavior change eHealth programs may be more effective by simulating a therapist’s support of a working alliance, internalization of motivation, and managing lapses.

**Methods:**

We show how the model is derived from theory and its application to *Endre*, a fully automated smoking cessation program that engages the user in several “counseling sessions” about quitting. A descriptive case study based on tools from the intervention mapping protocol shows how each therapeutic process is simulated.

**Results:**

The program supports the user’s working alliance through alliance factors, the nonembodied relational agent *Endre* and computerized motivational interviewing. Computerized motivational interviewing also supports internalized motivation to quit, whereas a lapse management component responds to lapses. The description operationalizes working alliance, internalization of motivation, and managing lapses, in terms of eHealth support of smoking cessation.

**Conclusions:**

A program may simulate working alliance, internalization of motivation, and lapse management through interactivity and individual tailoring, potentially making fully automated eHealth behavior change programs more effective.

## Introduction

“Black boxes,” or poorly described programs, have long been a criticism of the eHealth field [1-4], and effective program components across individual interventions are still largely unknown [5]. To address this problem, assumed mechanisms should be adequately described and put in a theoretical context [6]. This would build well-founded hypotheses for active program ingredients. Theoretically founded hypotheses may be especially useful in fully automated programs because automation standardize the therapy that is given. The standardization allows for program elements to be described in detail and investigated empirically, free from human variations and with a large degree of reliability. Investigating eHealth programs in light of counseling theories may increase our understanding of how such programs work [6]. In this paper, we will break down the components of an eHealth program and discuss its potential for interactivity and tailoring in terms of common concepts from face-to-face counseling. We hypothesize that simulating the therapeutic processes of supporting a working alliance [7,8], internalized motivation [9], and lapse management [10] simultaneously may be important to optimize behavior change.

According to Riley and colleagues, traditional health behavior change theories are static and linear in nature, and therefore, do not take advantage of the potential involved with interactive eHealth interventions [6]. eHealth interventions are not necessarily static or linear, as they can follow individual users and respond with tailored output to their immediate and previous responses. This enables dynamic adjustment of the intervention delivered, and theories from face-to-face counseling may therefore be more suited to understand eHealth interventions’ effective ingredients [6]. In this paper, therefore, we examine *Endre*, a fully automated program for smoking cessation that uses a fictional “therapist” to conduct tailored “counseling” sessions with the user.

**Figure 1:**
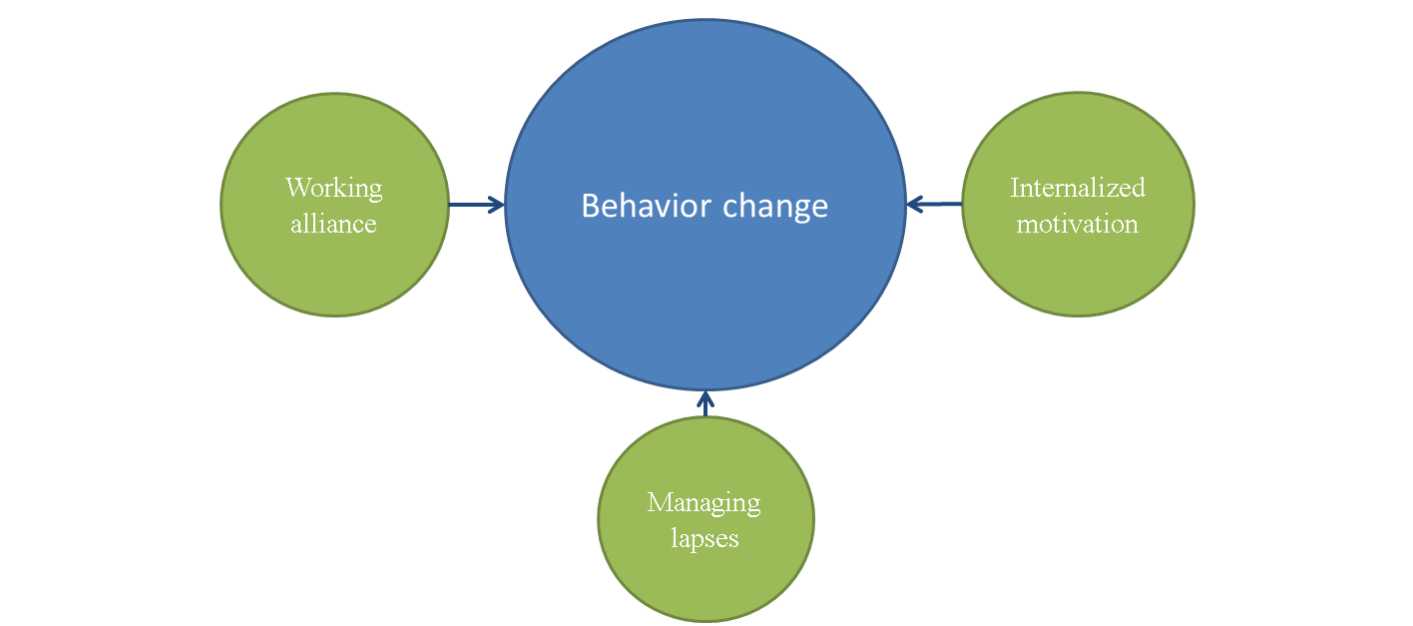
Different therapeutic processes affect behavior change differently.

Within eHealth-assisted behavior change, there is a growing interest in the concept of a working alliance [11-22], which is found essential in face-to-face counseling [7,8]. The alliance is commonly defined as an emotional bond, as well as agreement on task and goal [7]. It can also be described as therapist processes—such as empathy, warmth, and genuineness, establishing a collaborative framework and offering support and guidance [23]. A strong alliance facilitates client processes that are central to therapy-assisted behavior change, such as expectancies, intentions, motivation, hope, openness, trust, commitment, satisfaction, and a changing view of the self [23]. It may be possible to develop a working alliance to a fully automated program [12,21,22], but so far, there are only a few examples of programs designed to support a working alliance [11,12]. Likewise, motivational interviewing (MI) [24] is considered an effective method to motivate client change in counseling [25]. The effectiveness of MI has been linked to its ability to influence 3 basic psychological needs, including competence, relatedness, and autonomy [26,27]. By supporting these needs, external motivation, a weak form of motivation characterized by performing an activity to gain an external reward or avoid an external punishment, can become internalized. This means the activity is performed because the individual accepts it as an important step toward a personally valued goal [26], improving self-regulation, performance, and persistence [9,27,28]. Although MI is often mentioned as one of several methods in eHealth programs [29-33], only 2 report MI as a main method applied extensively [32,33]. Finally, behavior change is difficult, and even when an individual is motivated and the change is going well, he or she still needs to avoid lapses or setbacks in behavior. If a lapse should occur, the individual needs to react constructively to avoid a complete relapse. Teaching people how to prevent a lapse from becoming a relapse (lapse preparation), and helping them manage lapses (lapse management), is thus important when implementing behavior change [10]. Lapse preparation and lapse management have previously been applied to fully automated eHealth programs [31,34-37], but its effect has not been documented. Each therapeutic process has a unique contribution to the user’s change process. Supporting internalization of motivation gives the user strength and persistence in upholding the change [9,27,28]. Helping the user manage lapses keeps him or her from resuming the old behavior after a setback. Finally, supporting a working alliance makes a positive therapy outcome more likely [7,8] (Figure 1).

No published description exists, as far as we know, of a program supporting all 3 processes simultaneously, as proposed in the theoretical model in Figure 1. The aim of this paper is therefore to illustrate this model through a case study of *Endre*, a fully automated smoking cessation program, and to forward a hypothesis of these 3 therapeutic processes as important eHealth elements. We use a focused, descriptive analysis to conceptualize the translation from theory to intervention. The analysis is based on a modified intervention mapping protocol [38], which is a framework for designing and planning health promotion interventions through a taxonomy of mapping tools that can be used to code intervention contents. We use the steps that target process theory, methods, and design integration (steps 2-4) to focus on the 3 therapeutic processes that constitute the working hypothesis of *Endre*. This paper therefore also exemplifies the use of intervention mapping as an approach ideally suited to investigate potentially important elements in the “black box” of eHealth programs.

## Methods

### The Case: Endre

*Endre* is a fully automated eHealth program for smoking cessation that has evolved from the third author’s experience with the smoking cessation program *Happy Ending* [34]. *Endre* has some of the same basic structures as *Happy Ending*. It uses tunneling [18,39], has both pull (Web page) and push elements (e-mails and short message service [SMS] messages), and delivers program materials through the “voice” of a nonembodied relational agent [11]. Importantly, lapse management (with Marlatt’s cognitive behavioral model of relapse prevention [10] as methodological counterpart) is a central component of both *Endre* and *Happy Ending.* However, as opposed to *Happy Ending*, which in addition to lapse management consisted of a large number of theoretical and methodological underpinnings [34], the content of *Endre* is centered on 2 other theoretical concepts: internalized motivation (with MI as the methodological counterpart [26,27]) and working alliance (with *alliance factors* [13] as the methodological counterpart).

*Endre* consists of 26 tunneled [18,39] Web sessions. On registration, users provide their mobile phone number and e-mail address, which prompts receipt of an automatically generated e-mail with a username and password. After the program starts, the user goes through 10 days of preparing to quit with one new session each day, followed by their quit day, which is scheduled on the 11th day. The user must confirm a quit attempt before the program moves on to the follow-up phase. In the follow-up phase, the user gets one new session the first 3 days, then 2 new sessions every week for the first 4 weeks, and finally one new session a week for the last 4 weeks. The program ends 8 weeks after the cessation day. Automatically generated e-mails give the user access to each new session through a link. The links are time based, they lead to today’s session for that individual user, and one cannot access earlier sessions by clicking on old links. If a user rarely logs on, he or she will only receive the most important missed sessions. An overview of the themes for each session can be viewed in Multimedia Appendix 1.

*Endre* provides no additional human support. Most sessions involve user interactivity, requesting input from the user (see screenshots below for examples). We anticipate that an adult, typical user with average reading abilities may spend 4-6 minutes on each session. The user receives synchronous and immediate feedback on input. The lapse management component of *Endre* is based on the lapse management component in *Happy Ending* [34] and consists of daily SMS messages that are sent out to users who have quit, asking them if they have been smoke free that day. If the user reports a lapse, he or she gets access to a special, Web-based session intended to help the user recover from the lapse (Multimedia Appendix 2). This special session can be accessed whenever and for as many times as necessary.

### Analytic Procedure

We describe how a counselor’s support of a working alliance, internal motivation, and lapse preparation and management are simulated in *Endre* by using selected steps from the intervention mapping protocol (steps 2, 3, and 4) [38]. Intervention mapping is well suited for describing process simulation because it can be applied to understand the program construction. Furthermore, the necessary information for an intervention mapping analysis was readily available, as *Endre* was developed using intervention mapping. Intervention mapping is conventionally used to describe everything in a program [29,30,40-50]. Contrarily, we use it in a focused way to describe only the elements that are relevant to our hypothesis of important program elements. The intervention mapping tools are thus used for an analysis consisting of 2 parts: First going from general therapeutic process to theoretical operationalization suiting the context of this program; and second, going from theoretical operationalization to simulation in specific program elements.

First, we describe how supporting a working alliance, internalized motivation, and lapse preparation and management are operationalized in *Endre* ’s theoretical change model (step 2 in intervention mapping [38]). In the change model, the changes necessary to quit smoking by means of *Endre* are described and displayed in a matrix. In the intersecting cells of the matrix, the operationalization of each therapeutic process is described in a list of change objectives. That is, each change objective shows how one aspect of one of the therapeutic processes is operationalized for the purpose of the intervention (that the user quits smoking and stays smoke free with *Endre*) and its context (a fully automated program). An analytic text accompanies the change model to describe how the 3 processes are represented in the change model. The change model that was used for the development of *Endre* (Multimedia Appendix 3) is simplified to highlight the 3 therapeutic processes, and we use sequential numbering of the change objectives instead of conventional intervention mapping-labeling [38] to improve readability outside of the intervention mapping community. The change model operationalizes the abstract and general therapeutic processes. It is therefore the first part of the analysis toward the processes’ simulation.

After showing how supporting a working alliance, internalized motivation, and lapse preparation and lapse management are operationalized through change objectives, we describe how the 3 therapeutic processes are simulated through specific program elements (steps 3 and 4 in intervention mapping [38]). The program elements result from combining change objectives with theoretical methods for inducing change (eg, MI, modeling). This second part of the analysis takes the (theoretical) operationalizations of the 3 therapeutic processes and makes them into (practical) simulations through specific program elements.

## Results

### Operationalization of the Therapeutic Processes in Endre

The operationalization of the therapeutic processes can be viewed in the change model matrix (Table 1). In the matrix, sub-behaviors in quitting, or performance objectives, are crossed with theoretical constructs, or personal determinants *,* believed to be causing or influencing the behavior. Each therapeutic process is represented within the model either as a personal determinant or a performance objective. The personal determinants and performance objectives intersect in cells containing change objectives *,* which specify how each personal determinant must change for the individual to be equipped to do each performance objective.

Working alliance and internalized motivation are operationalized as personal determinants, whereas behavior maintenance through lapse preparation and lapse management is operationalized as a performance objective. Having a working alliance to the program is not a necessary psychological process for quitting smoking in general. It might, however, be an important process for quitting smoking with the help of *Endre*, if one assumes that a successful simulation of supporting a working alliance can have the same benefits for therapy outcome in a fully automated program as it has in face-to-face counseling [7,8]. Though a working alliance can be an important psychological process for quitting smoking with *Endre*, internalized motivation is an important psychological process for succeeding in quitting smoking at all. In the model, internalized motivation is separated into the underlying personal determinants relatedness, competence, and autonomy; the 3 “needs” that influence the internalization of motivation [9]. Competence is itself separated into 2 personal determinants: skills and self-efficacy. As with competence, relatedness is also separated into 2 personal determinants: relatedness to social network and working alliance. Working alliance, or relatedness to the program, is included under relatedness because a positive counseling relationship can also support the client’s (or user’s) need for relatedness [27]. In contrast, behavior maintenance through lapse prevention and lapse management is operationalized in the change model as a performance objective, meaning that managing lapses in a constructive way is considered an important subgoal for succeeding in quitting smoking. The change objectives belonging to each therapeutic process is the operationalization of that process for the purpose of this program.

**Table 1:** Modified change model.^a^

Performance objectives	Personal determinants				
	Internalized motivation (therapeutic process 2)				
	Relatedness		Competence		Autonomy
	Working alliance (therapeutic process 1)	To social network	Skills	Self-efficacy	
1. Decide to quit smoking and plan how to do it.	1. Experience the program as a social actor [11]. 2. Experience the program as accessible, helpful, empathic, and trustworthy [13]. 3. Be aware of one’s influence on program content [13]. 4. Understand how to use the program and do the exercises [13].	5. Make a public commitment to the quit attempt. 6. Choose a “support person” from one’s personal network.	7. (1) Identify personal smoking cues and (2) be able to detect smoking urges early. 8. Make an action and coping plan for the quit attempt. 9. (1) Identify one’s high-risk situations, and (2) make an action and coping plan for handling them [24].	10. Believe it to be possible to quit smoking and stay smoke free [13]. 11. Be confident in one’s ability to execute the action and coping plan [24].	12. Commit personally to the quit attempt and know one’s personal reasons for doing so [24]. 13. Decide whether or not to (1) make a public commitment and (2) engage a “support person.” 14. Choose how to make the quitting plan (by oneself or a more guided version) [24]. 15. Combine the advice of the program with one’s own style and preferences [13].
2. Initiate the quit attempt and stay smoke free for the first 3 days.	16. Experience the program as: (1) responsive, sensitive, and adjustable for emerging needs and (2) suiting one’s own preferences and style [13].	17. Ask the “support person” for practical assistance and emotional support as needed [51].	18. Implement action and coping plan. 19. Get rid of remaining cigarettes and smoking accessories. 20. Withstand cravings and cope with withdrawal symptoms.	21. Be confident in one’s ability to stay smoke free the first 3 days [24].	22. Revise the action and coping plan if needed. 23. Decide whether or not to get rid of remaining cigarettes, or whether to make the cigarettes less accessible. 24. Decide to what degree, when and how the “support person” is needed.
3. Establish a smoke-free lifestyle (from day 4 and onward).	25. Continue with the program for as long as needed, even after a period of program disengagement (“rupture prevention and repair”) [13].	Same as the above (change objective 17).	26. Identify and counteract thought patterns that could lead to a (re)lapse [52]. 27. Follow plans for high-risk situations.	28. Be confident in (1) one’s ability to continue being smoke free, and (2) one’s ability to stay smoke free in the long run.	29. Attribute success in the cessation attempt internally.
4. Maintain the behavior by managing lapses constructively (therapeutic process 3).	Same as the above (change objectives 16 and 25).	30. Explain the difference between a lapse and a relapse to significant others to gain their continued support.	31. Know the difference between a lapse and a relapse, and how to recover from a lapse. 32. Get rid of any spare cigarettes after a lapse. 33. Resist new urges to smoke.	34. Be confident in one’s ability to continue with the quit attempt after a lapse.	35. Know that whether or not to remain smoke free is a matter of one’s own choice.

^a^Every cell specifies the theoretical operationalization of one (or several) therapeutic process(es).

### Simulation of the Therapeutic Processes in Endre

In this section, we describe how the therapeutic processes are simulated in *Endre*. For each therapeutic process, we present program elements that are involved in the simulation and describe the methods that are used. To support working alliance we adapted MI [24] to a computerized “counselor” who delivers all program material through what we refer to as computerized motivational interviewing (cMI). The “counselor” is called *Endre*, which has a double meaning in Norwegian, being a man’s name, as well as literally meaning “to change.” Internalized motivation is primarily supported through cMI, whereas behavior maintenance is strengthened with a psycho-educative session before the quitting day, as well as a special Web-based session that is made accessible if the user reports a lapse. If the user experiences several lapses, this is recognized by *Endre*, and the content of the session is adjusted accordingly.

### Simulation of Working Alliance Support

**Figure 2:**
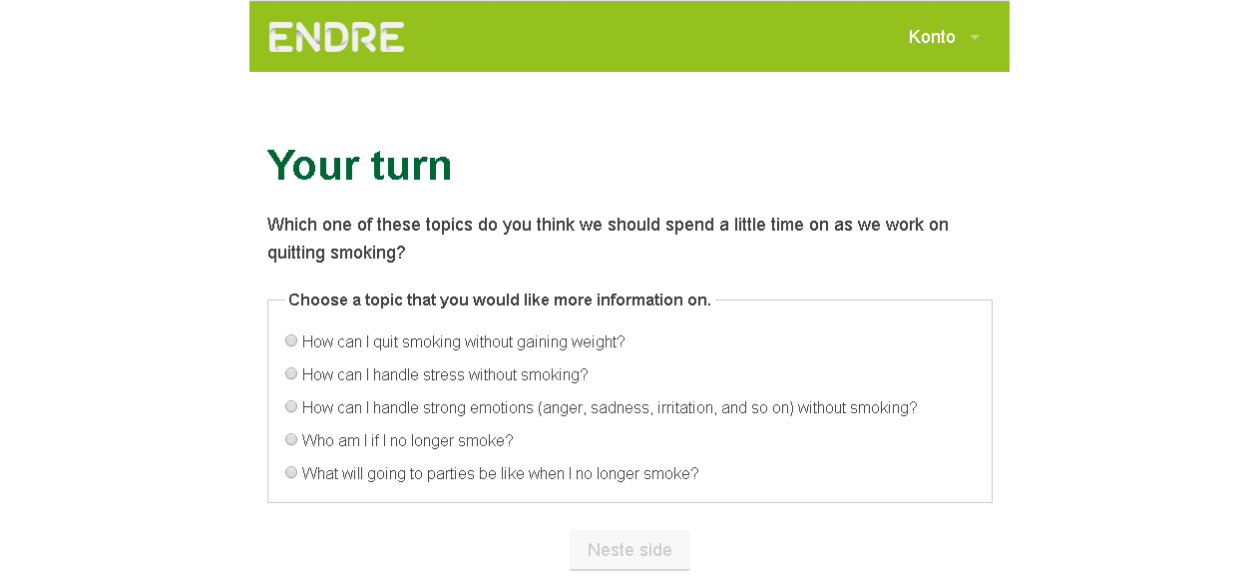
Choosing a topic (“negotiating” goals).

**Figure 3:**
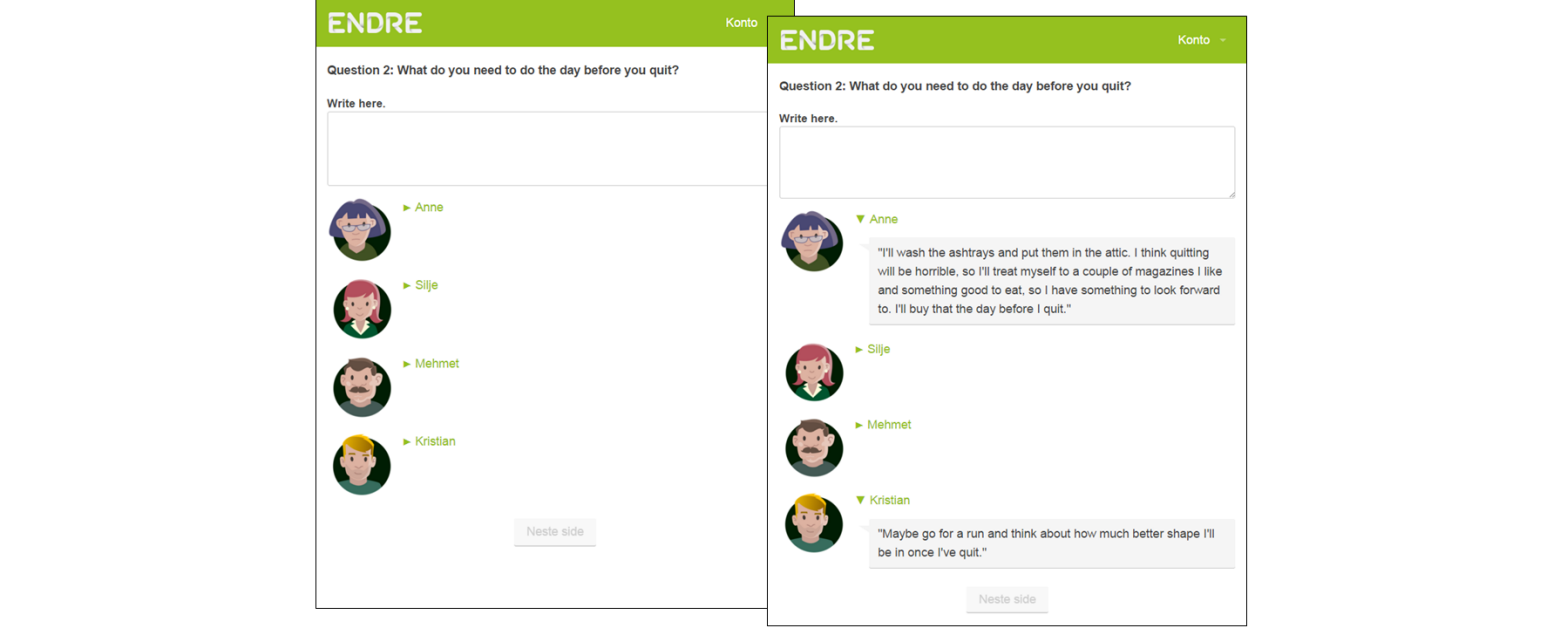
The 4 “quitters” demonstrate how to do the program exercises and model how to combine Endre’s advice with one’s own personal style.

**Figure 4:**
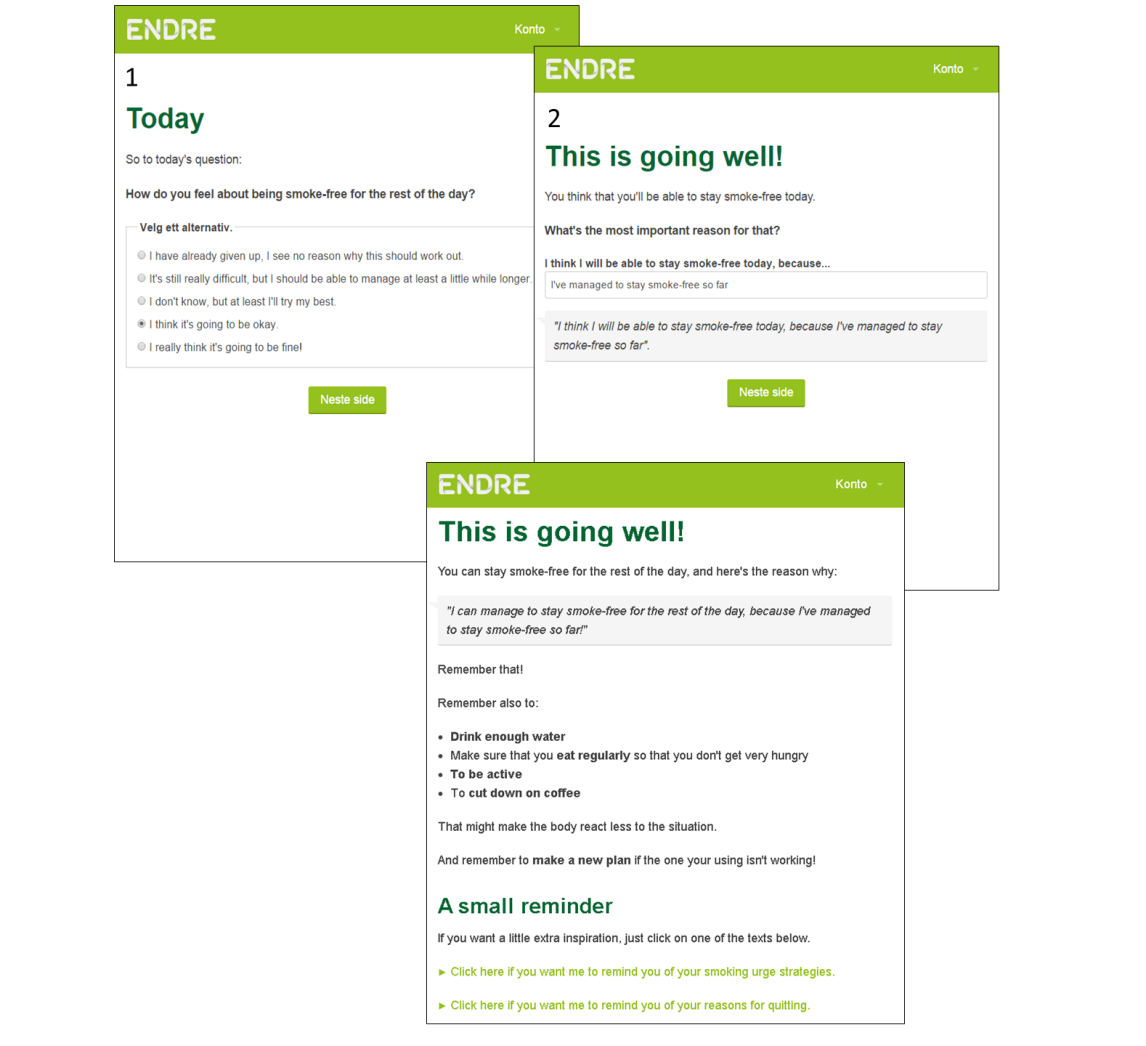
Eliciting self-efficacy change talk through a confidence ruler.

**Figure 5:**
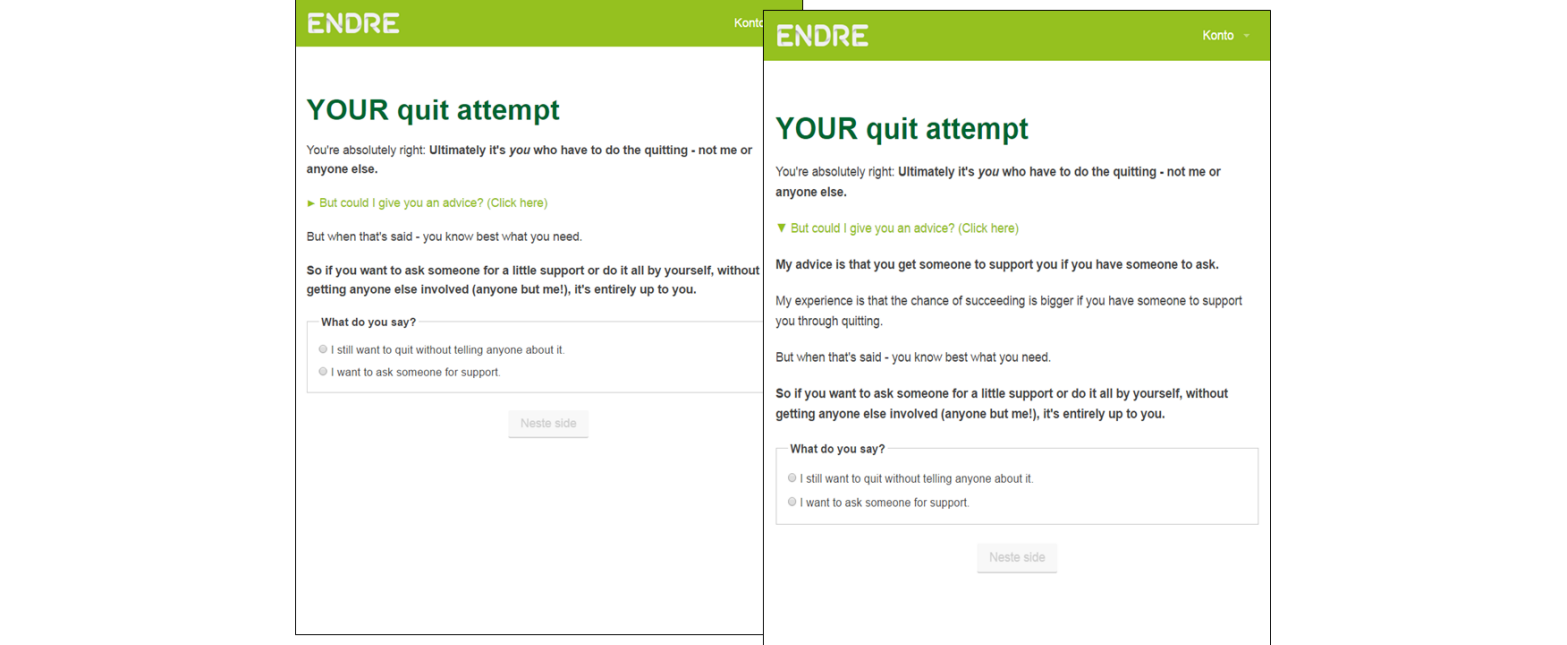
Endre has asked the user to choose a “support person” for her quit attempt, and the user has answered that he or she wants to quit without any help.

**Figure 6:**
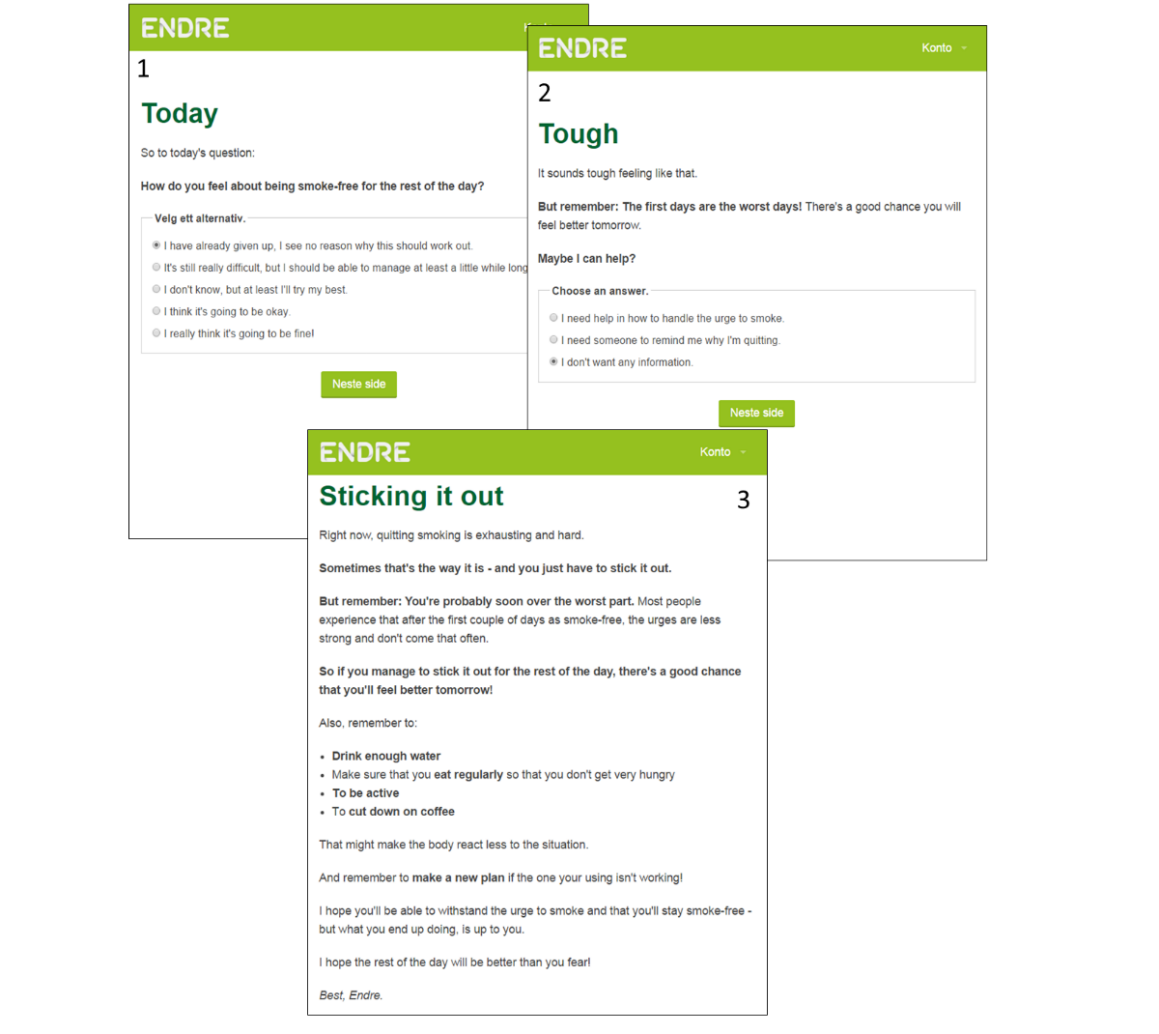
Handling sustain talk and discord.

Working alliance is supported in program elements using a nonembodied relational agent [12], cMI (Multimedia Appendix 4), and dynamic tailoring [42] to convey alliance factors [13]. For the users to experience the program as a social actor [11] (change objective 1) that is accessible, helpful, empathic, and trustworthy [13] (change objective 2), the relational agent [12] *Endre* is used throughout the program. *Endre* is a nonembodied, text-based relational agent that simulates a “counselor” the user “communicates” with. Some key attributes of *Endre* can be found in Textbox 1, and examples of how “he” is represented in the program can be found in Figures 2-6.

**Attributes of the relational agent Endre.**

Uses first person tense.

Introduces a new topic for each session.

Asks questions and reflects answers empathically [24].

Uses appropriate greetings and farewells according to time of day [12].

Uses humor [12].

“Remembers” earlier conversations by explicitly referring to them or implicitly adjusting program content.

To further support a working alliance, users are allowed to influence the program content [13] (change objective 3). This is a way of “negotiating” goals [13] and is done in the first session (Figure 2). After *Endre* has presented the program plan, the user is asked to choose a topic he or she considers important when quitting. On the subsequent page, *Endre* assures the user that “he” will make time for this topic during the course of the program. The user’s topic is visited 2 times during the program.

To build a working alliance to the user, it is also necessary for him or her to receive guidance in how to use the program [13] (change objective 4). *Endre* provides guidance to the user, for example by explaining how new sessions are made available and how the user can log onto them. In addition, new program exercises are demonstrated by four fictional “quitters” (Figure 3).

Working alliance is further strengthened if the program is experienced as responsive, sensitive, adjustable for emerging needs, and suiting one’s own preferences and style [13] (change objective 16). To address this, *Endre* has a *flexible session manager* (Textbox 2) that adjusts the total number of sessions to user behavior. This means that a user who does not log on to the program every time a new session is available will only receive the most important sessions, limiting the total number of sessions for that particular user.

**Flexible session manager.**

Ensures that a user who has missed several sessions receives the most important session of the ones he or she has missed.

A user that seldom logs on will only get the most important sessions of the program.

We developed a set of rules that decides what session the user will get next (ie, the most important sessions), based on:

the program plan,

which sessions the user has already logged on to,

rules that categorize the sessions as either high priority (all users must go through these) or low priority (the user only receives these if he or she has done the high-priority sessions thus far).

A user who has missed several sessions first receives those that are categorized as high priority.

If the user has logged on to all high-priority sessions, he or she receives low-priority sessions that address (in the following order): skills, self-efficacy, relatedness, and autonomy.

A final program aspect supporting working alliance is a “mini motivation intervention” (Textbox 3), consisting of SMS messages and intended to prevent program disengagement (alliance “rupture” [13]) (change objective 25).

**Mini motivation intervention.**

Before quitting day:

If the user misses one session, nothing happens.

If the user misses 2 sessions, he or she gets an SMS message from *Endre*, reminding him or her to log on.

If the user misses 3 sessions, he or she gets an SMS message where *Endre* normalizes having second thoughts and recommends logging on to the program.

If the user misses a fourth session, nothing happens.

If the user misses a fifth session, he or she gets a final SMS message where *Endre* appeals to the “healthy part” of the user to log on.

After quitting day:

After quitting day, there is no intervention if the user does not log on to the Web page.

A part of the lapse management system is that the user every evening receives an SMS message, asking if he or she has been smoke free. If the user does not answer the SMS message, he or she will receive up to 3 extra SMS messages encouraging him or her to answer.

### Simulation of Internalized Motivation Support

Internalized motivation is achieved through *Endre* strengthening the user’s autonomy, competence, and relatedness [9]. Relatedness is partly supported through building a working alliance between the user and the program, as described in the previous section. The other part of relatedness, relatedness to social network, is strengthened through helping the user find support in the people surrounding him or her. This is done by advising the user to recruit a “support person” from his or her social network (change objective 6), advising him or her to make the quit attempt public (change objective 5), and guiding the user in how to make their “support person” have the greatest positive impact on his or her quit attempt (change objective 17). Figure 5 is from the session where *Endre* advices the user to choose a “support person,” showing what happens when the user does not want to follow *Endre* ’s advice. Another way in which *Endre* supports the user’s relatedness to his or her social network is effectuated if the user reports a lapse after he or she has quit. *Endre* then asks if this lapse may affect the user’s relationship to his or her social network. If the user answers yes, *Endre* offers help to ensure the social network’s continued support for the quit attempt (change objective 30). All advice is given using cMI (Multimedia Appendix 4) and dynamic tailoring [42].

Autonomy is supported in program elements using cMI, dynamic tailoring [42], and modeling [38]. One way *Endre* supports autonomy is by asking for permission before giving any information or advice (Textbox 4). This is a way of acknowledging that the user chooses what information to receive. Asking for permission is relevant to change objectives 13, 15, 23, and 24.

**Asking for permission.**

*Endre* requires one of two actions from the user before giving any information or advice:

Hide/show text: The user can choose whether or not to click on the question (eg, “do you want me to tell you about…”) to reveal the information. An example can be viewed in Figure 3.

Question + multiple choice yes/no: The user must answer yes or no when *Endre* asks for permission to give information or advice; if the user answers yes, the information is revealed on the next page. For example, one session starts by *Endre* introducing today’s topic, and then asking the user whether he or she thinks this sounds okay. If the user answers yes, the session continues. If the user answers no, the session is ended.

A second way in which autonomy is supported is through handling *sustain talk* (reasons for smoking) and *discord* (dissatisfaction with therapy) [24] respectfully. Sustain talk and discord may be expressed by the user at select places in the program through multiple-choice alternatives. The fact that expressing sustain talk or discord is allowed (even when it goes against the program) communicates respect for the user’s autonomy. If sustain talk or discord is expressed, *Endre* repeats the user’s feelings empathically, and then, depending on the situation, asks more questions, normalizes, offers help, or changes the topic [24]. Handling sustain talk and discord is relevant to change objectives 13, 15, 23, 24, and 35. An example of how sustain talk or discord may be expressed and how it is handled can be seen in Figure 6. This is from the user’s second day as smoke free. On page 1, *Endre* asks the user how he or she feels about staying smoke free for the rest of the day. The example shows the user choosing the statement representing the lowest degree of self-efficacy; so low that it qualifies as sustain talk. On page 2, *Endre* offers help. The user chooses that he or she does not want any help; this can be seen as dissatisfaction with the program, or discord. On page 3, *Endre* reflects empathically and normalizes the user’s feelings.

A third way *Endre* supports the user’s autonomy is through eliciting and reflecting *change talk*, that is, talk arguing toward change [24] (change objective 12). Change talk is the user’s autonomous reasons and capacities for quitting and is requested throughout the program. *Endre* repeats the user’s change talk and sometimes elaborates on it. For example, in one session, *Endre* asks the user for his or her most important reason for wanting to become smoke free (eliciting change talk). *Endre* repeats the user’s most important reason on the next page (reflecting change talk). Asking for permission, handling sustain talk and discord, and eliciting change talk is achieved through cMI and dynamic tailoring [42], and details of these applications can be viewed in Multimedia Appendix 4.

A fourth and final way in which *Endre* supports autonomy is through modeling [38]. In the program, 4 fictional “quitters” model autonomy by illustrating how to combine the advice of the program with one’s own style and preferences (change objective 15). The 4 “quitters” are of different gender, age, socioeconomic status, cultural background, and *smoking profiles* [53]. The “quitters” answer *Endre* ’s questions and tasks in ways that suit their situation and personality. An example of this application can be viewed in Figure 3. This screenshot is from the session for making a cessation plan, where *Endre* asks the user what he or she needs to do the day before quitting. By clicking on the names of the 4 fictional “quitters,” the user may read “their” answers.

Autonomy is supported through asking for permission before giving advice, handling sustain talk and discord respectfully, eliciting and reflecting change talk, and modeling how to combine the program’s advice with one’s own preferences and style. Competence is supported through increasing the user’s quit-related skills and increasing his or her self-efficacy for quitting. Skills can be acquired through the general information and advice that *Endre* gives, as well as through program exercises. For example, before quitting day, *Endre* asks the user to spend a few days thinking about what precedes his or her smoking—what are his or her *smoking cues*. After a few days, *Endre* asks the user for these smoking cues. This teaches the user to be attentive to what triggers the urge to smoke. The advices and exercises that *Endre* gives are based on self-monitoring of behavior, counter-conditioning, active learning, goal setting, planning coping responses, and implementation intentions [38], always communicated using cMI (Multimedia Appendix 4).

Whereas skills are supported through information, advice, and exercises, self-efficacy is supported through cMI techniques, in combination with dynamic tailoring [42]. The user’s self-efficacy is strengthened through “confidence rulers” [24,32]. An example of this application can be found in Figure 4. These screenshots are from the same session as the ones in Figure 6, but showing what happens when the user answers differently. In this example, the user chooses the statement reflecting a quite high degree of self-efficacy. On page 2, *Endre* asks the user to justify why he or she chose that statement over a statement representing a lower degree of self-efficacy. The user types in his or her answer, and on page 3, this statement is reflected back to him or her. The user has argued for change and had the argument reflected back, amplifying the effect [24].

Self-efficacy is also strengthened through affirmations [24], that is, compliments on the user’s strengths and accomplishments. For example, in one session, the user is asked if he or she has tried quitting before. If the user answers yes, *Endre* replies that this is a good thing, because the user then has experience that he or she can use to increase the chances of succeeding this time. Turning previous quitting experience into something positive is a way of providing affirmation, supporting self-efficacy, competence, and internal motivation.

### Simulation of Lapse Preparation and Lapse Management Support

**Figure 7:**
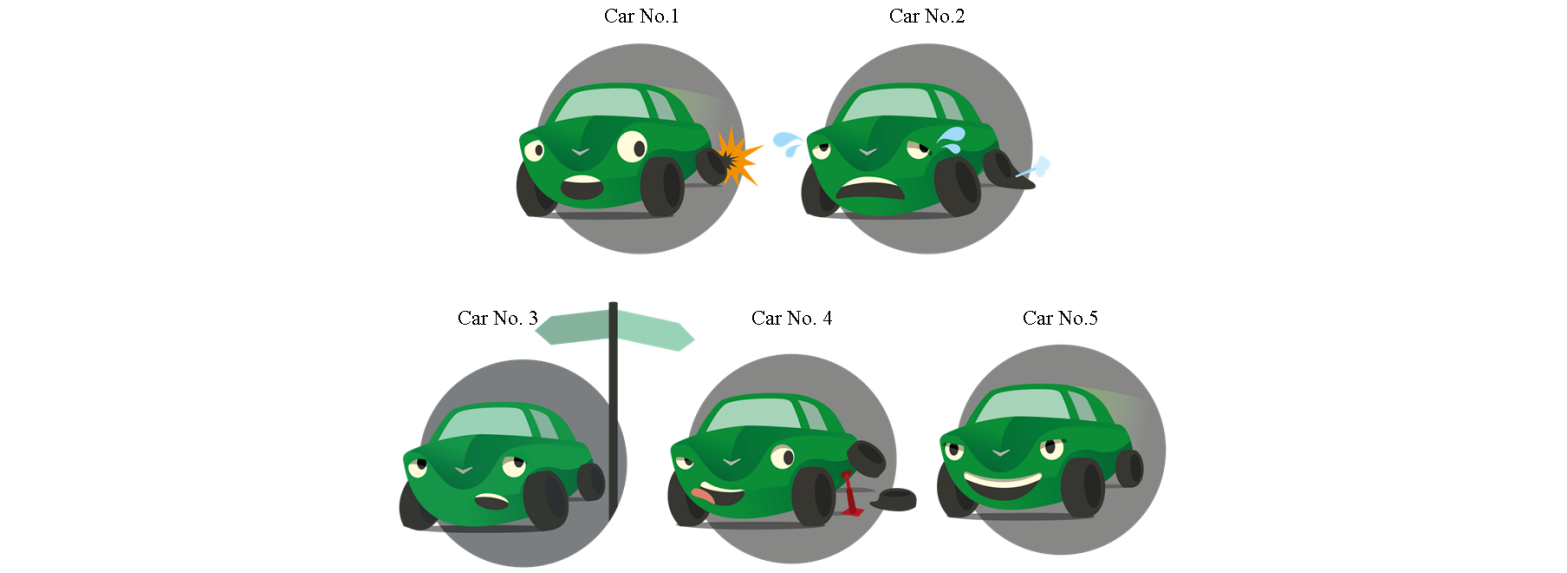
Visual analogy for lapsing and resuming the quit attempt.

Behavior maintenance is supported through a psychoeducative session before the user’s quit day and a lapse management component after he or she has quit. First, a psychoeducative session on lapses and relapses prepares the user to respond constructively in case of a lapse (change objectives 31 and 35). In this session, a car puncturing a tire is used as a visual analogy [38] for lapsing and relapsing. The cars can be seen in Figure 7. Car no.1 illustrates the lapse (puncturing the tire), car no.2 illustrates a relapse (giving up and succumbing to negative emotions), car no.3 shows the process of choosing, car no.4 is acting to resume the quit attempt, and car no. 5 illustrates being smoke free again.

**Figure 8:**
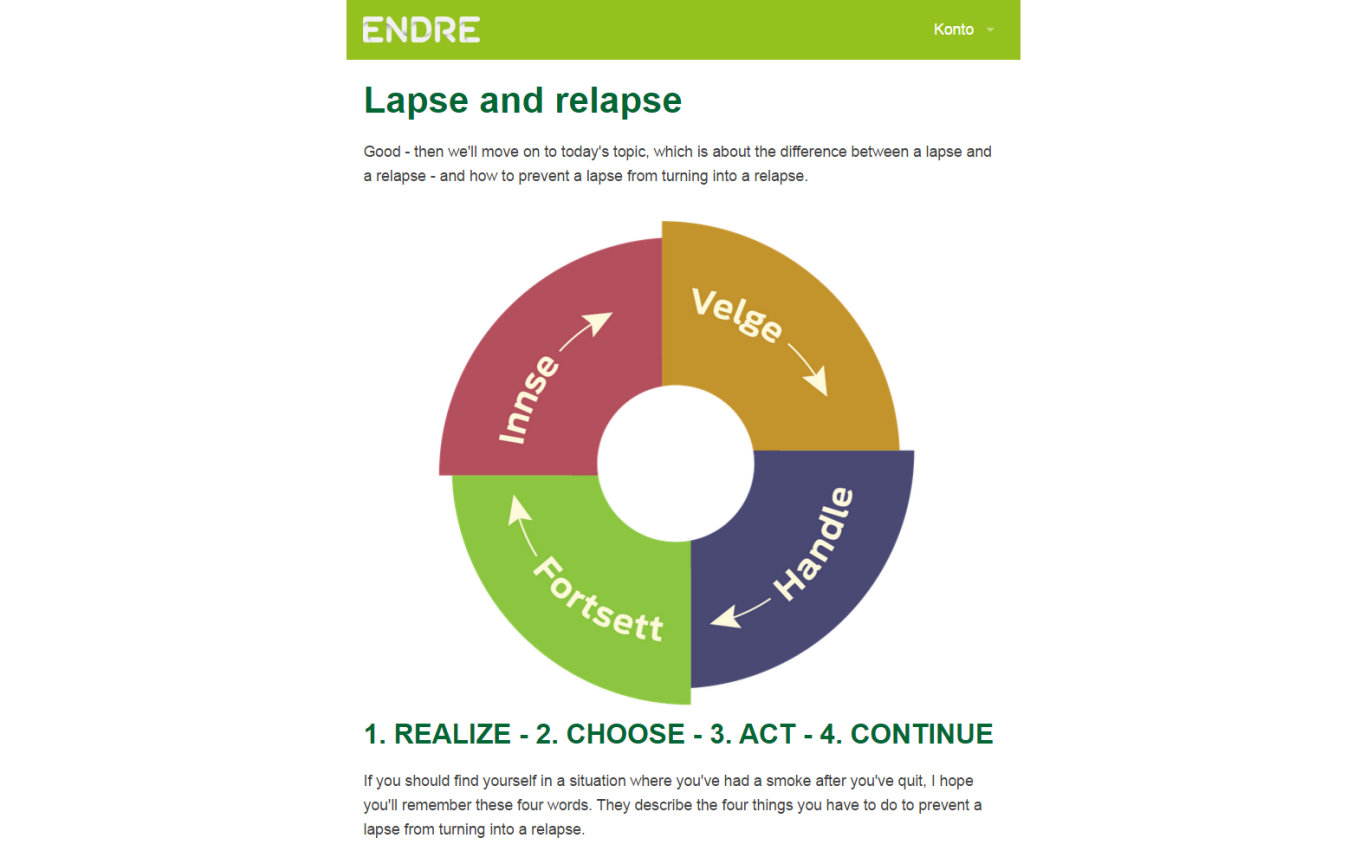
Advance organizer of returning to the quit attempt after a lapse (from top left section): realize (“innse”), choose (“velge”), act (“handle”), and continue (“fortsett”).

In the preparatory session, the user is also presented with an advance organizer [38] of the process of becoming smoke free again after a lapse. The advance organizer has the shape of a circle (Figure 8) displaying the self-regulation loops [54] that can help the user back to being smoke free. First, realize that you are smoking (“innse”), then choose: Keep smoking or keep quitting (“velge”), then act to become smoke free again (“handle”), and finally continue with being smoke free (“fortsett”). The information is given with cMI (Multimedia Appendix 4).

**Figure 9:**
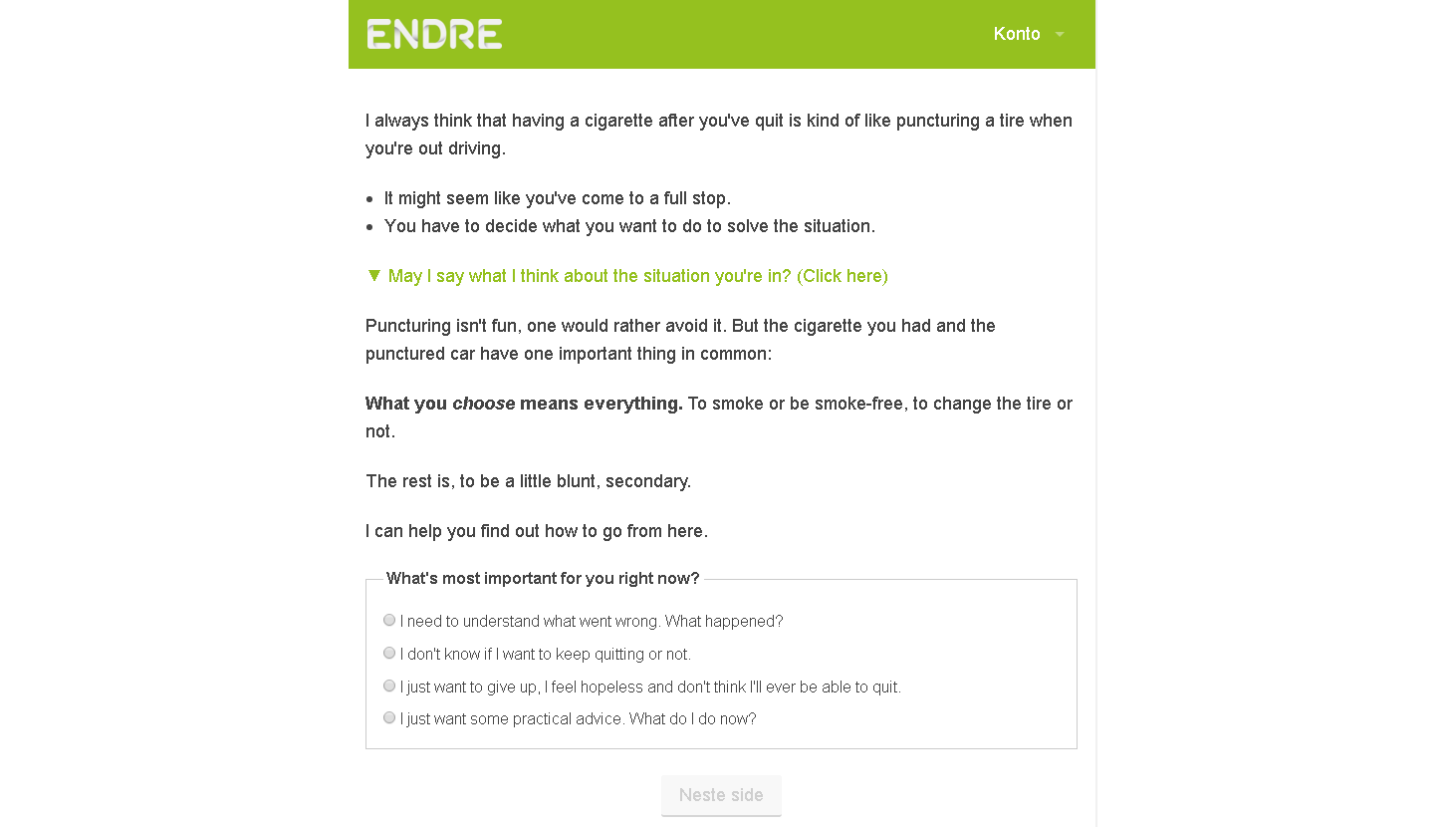
From the lapse management session: the user is unsure of what to do and is asked what topic to begin with.

Following up on the preparatory session on lapses and relapses is a lapse management component which is effectuated after the user has confirmed a quit attempt. Every day, the user receives an SMS message asking if she is still smoke free. If the user answers yes, another SMS message compliments the user’s accomplishment. If however the user answers no, he or she receives an SMS message with a link to a Web-based lapse management session. The user may access the session through the SMS message; if he or she does not log on via the SMS message, he or she receives the lapse management session when logging on to the program next time. The lapse management session helps the user make a choice, become smoke free again and learn from the lapse. When logging on to the Web-page, the user is first reintroduced to the car (Figure 7) and the circle (Figure 8). *Endre* then asks if the user has already decided what to do: keep quitting or keep smoking (Figure 9). If the user chooses to keep quitting, *Endre* guides him or her back to being smoke free, helps making a new plan on how to face a similar situation in the future without lapsing, and supports the user’s belief in his or her ability to stay smoke free. Figure 9 shows a screenshot from the lapse management session. In this example, *Endre* has asked the user if he or she knows what to do now, and the user has answered that he or she is unsure. On the next page, shown in the screenshot, the user may choose which topic he or she wants *Endre* to start with (the picture does not show the entire page). Asking the user what topic to start with is a way of asking for permission [24], strengthening his or her autonomy and supporting internal motivation. In addition, letting the user influence the program structure influences the working alliance positively [13]. This screenshot shows the main topics that are covered in the lapse management session: reattribution [10], ambivalence [24], the abstinence violation effect [10], and making a choice. Only users who express ambivalence or an abstinence violation effect when asked go through these topics. Multimedia Appendix 2 contains more information on the lapse management component, including a flow chart that shows the different ways in which this session may be built up. Some of the methods that are used are cMI (Multimedia Appendix 4), dynamic tailoring [42], reattribution [55], and cognitive restructuring [56].

## Discussion

### Summary Analysis

This case study illustrates our proposed theoretical model for eHealth behavior change interventions: simulating a counselor’s support of working alliance, internalization of motivation, lapse preparation, and lapse management simultaneously. The case, *Endre*, is a fully automated smoking cessation program where each session takes the form of a written “counseling session” between the user and the program. The program content and structure were analyzed using intervention mapping [38], illustrating the translation from theoretical model to intervention. The analysis shows that simulation of the 3 therapeutic processes is accomplished through a range of program elements. Working alliance [7,8] is supported through alliance factors [13], a nonembodied relational agent [12],cMI (Multimedia Appendix 4), and dynamic tailoring [42]. Internal motivation [9] is supported through cMI, dynamic tailoring, and modeling [38]. Finally, relapse is sought prevented through a psychoeducative session on lapses and relapses and a postquit day lapse management component.

By defining the components of a program and discussing its potentials for interactivity and tailoring in terms of concepts from face-to-face counseling, eHealth programs can be better understood [6]. This has implications both for program development and for the theoretical development of eHealth therapeutic process. In addition, by showing how the therapeutic processes of a program can be documented, from abstract concept through operationalization to simulation in specific program elements, we have demonstrated how intervention mapping used in a focused manner provides a compelling, interpretative approach to eHealth case studies. The value of such an inquiry for future empirical investigation is substantial: If the intervention should prove not to be effective, this may be because the identified theoretical processes are not sufficient for supporting behavior change or because the translation from theory to intervention elements was less than optimal.

**Figure 10:**
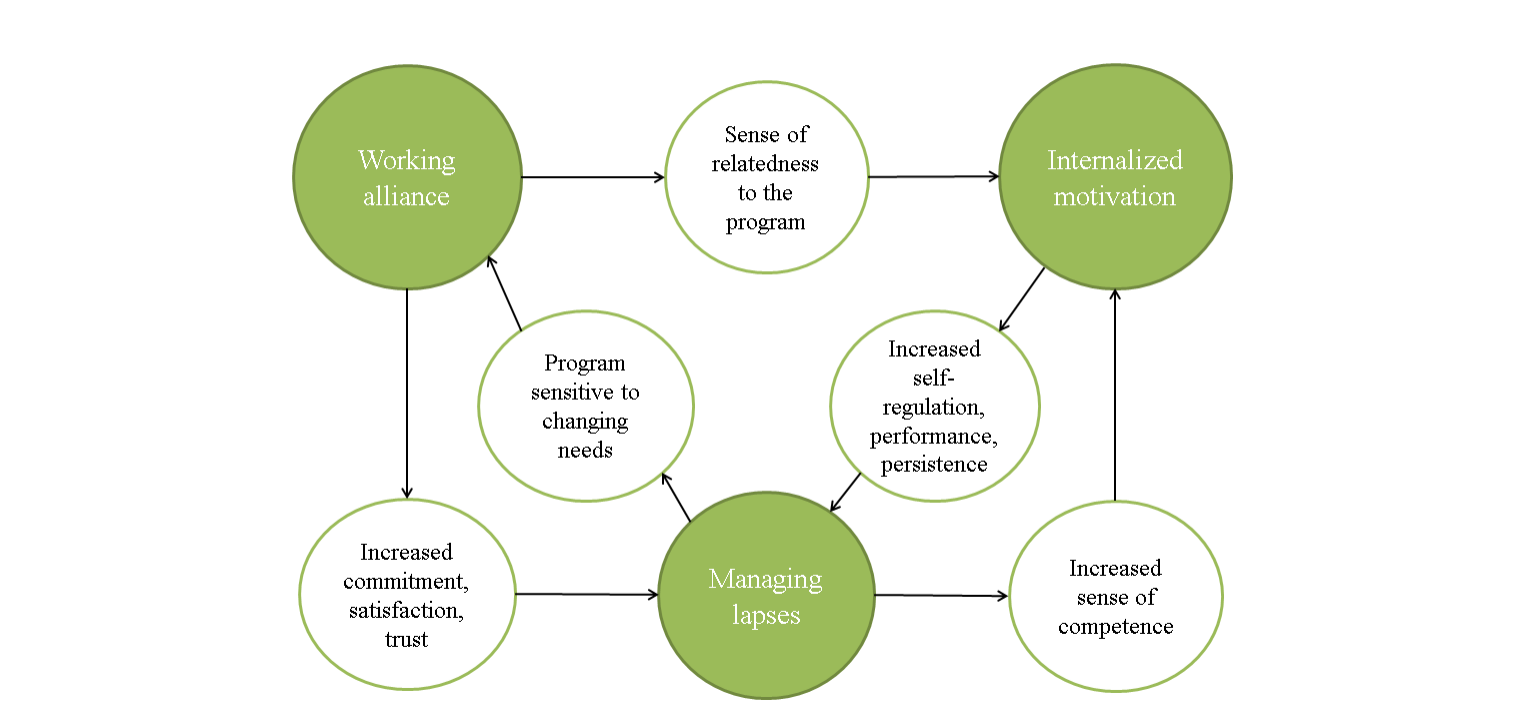
Hypothesized synergy effects of the 3 therapeutic processes.

The analysis of *Endre* suggests that the simultaneous simulation of each therapeutic process may result in a synergy effect. The operationalization in Table 1 reveals some of these potential interaction effects. The table visualizes that a working alliance is also a part of internalized motivation. When a working alliance to *Endre* is supported, this can influence the user’s need for relatedness, thus supporting his or her internalized motivation to quit [27]. In addition, Table 1 visualizes that a working alliance and internalized motivation (columns) cross behavior maintenance (row). This means that for *Endre* to succeed in helping the user manage lapses, he or she needs to have both a working alliance to *Endre* and internalized motivation to recover from a lapse, demonstrating that lapse management in a fully automated program can benefit from a strong working alliance and internalized motivation. A strong working alliance may enhance the effect of a lapse management program element through facilitating client processes such as commitment, satisfaction, and trust [23].This may increase the likelihood of the user staying with the program long enough to benefit from the lapse management therapy and trust the therapy that is given. At the same time, internalized motivation increases self-regulation, performance, and persistence [9,27,28] and may function as a buffer for future lapses. Should the user experience a lapse, a program that is supportive through that difficult period is likely to strengthen the working alliance by demonstrating sensitivity to the user’s changing needs [23]. Furthermore, if the user should succeed in overcoming the lapse it would also presumably increase his or her feeling of competence, again enhancing internalized motivation [27]. It seems therefore that simultaneous simulation of supporting a working alliance, internalized motivation, and lapse management may result in a mutual enhancement of each process. These hypothesized synergy effects are displayed in Figure 10.

Interaction can be assumed from the operationalization level, but the step to simulation also shows the many methods and program elements that support several therapeutic processes at once. For example, all program material is delivered by the relational agent *Endre* using cMI. A relational agent supports working alliance [12], and cMI supports both working alliance [25] and internalized motivation [26], but in different ways. *Endre* also uses cMI in the lapse management session, influencing all 3 therapeutic processes at once. Another example of a program element that support several therapeutic processes are the 4 “quitters,” serving both as guides in how to do the program exercises (supporting a working alliance) and as models in how to exercise autonomy in the quitting process (supporting internalized motivation). The fact that many program elements support several therapeutic processes at once implies that the effort needed to incorporate more than one therapeutic process in a program may diminish for each process included.

### Comparison with Prior Work

All 3 therapeutic processes have been applied to fully automated programs previously. Studies on working alliance have mostly been on relational agents [11,12]. *Endre* builds on this work, although applying a nonembodied, rather than embodied relational agent, allowing the user freedom to “create” aspects of the relational agent. To further support a working alliance, *Endre* also incorporates alliance factors [13]. “Endre” also builds on previous work in the application of MI [32,33] and use of lapse preparation and lapse management [31,34-37]. The most significant contribution of *Endre*, however, is simulating all 3 processes simultaneously, something that to the best of our knowledge has not been done before systematically in a fully automated eHealth program.

Finally, this paper extends earlier work using intervention mapping eHealth tools to present a focused descriptive analysis of chosen program elements. Papers that use intervention mapping usually follow the structure of the intervention mapping steps and reports on most of these [29,30,40-50]. Instead of giving a full account of the breadth of the program, this paper uses intervention mapping for a focused descriptive analysis to make an argument of possible important eHealth elements. The description is intended to be sufficiently deep to allow for further inquiry into the chosen elements. This application of intervention mapping represents a complementary approach to the standard use of the method, that is, instead of using intervention mapping as a purely descriptive tool, we use it as a normative tool to determine what elements *should* be present in the “black box” of eHealth programs.

### Limitations

Although comprehensive, the analysis presented here is a simplification of how the 3 therapeutic processes are simulated in *Endre*. Especially the social behavior of the relational agent, cMI, and dynamic tailoring are elements that are used in the entire program, and a full account was therefore not possible. Another limitation is that to highlight the 3 therapeutic processes, descriptive depth was chosen over descriptive breadth. In addition, *Endre* does not simulate the 3 therapeutic processes perfectly. A fully automated program neither has the flexibility nor the presence of an actual human being. Just as *Endre* is not a human counselor, cMI is not MI. But the program may nevertheless simulate these 3 therapeutic processes convincingly enough to derive some of the benefits they have in face-to-face counseling. It should also be noted that *Endre* only represents one way in which these therapeutic processes may be simulated. Thus, if *Endre* fails to be an efficient program, it may be because the therapeutic processes in a fully automated program are not successful in inducing change or because the simulation of the therapeutic processes in *Endre* was inadequate.

There are, of course, limitations to the type of program that *Endre* represents. First, not everyone who wishes to quit smoking may benefit from such a detailed program. In the first author’s clinical experience, some simply quit and do not wish to spend more time elaborating on the process. A participant in an earlier study conducted by the third author [57] actually experienced late night SMS messages asking whether she had been smoke free that day as smoking cues, creating a risk of (re)lapsing. *Endre* does make it possible for “unproblematic” quitters to move through many of the sessions rapidly, and the flexible session manager makes it possible to complete fewer sessions than what is in the full program. Nevertheless, it is a quite extensive intervention, communicating an expectation that quitting smoking is a process instead of a one-time action and requiring answers to daily SMS messages. Second, not everyone may wish to convey their thoughts with a program. Efforts to simulate a therapeutic setting aside, the therapy may still seem too artificial and ultimately unconvincing to the user. Alternately, the simulation may be too convincing, and sharing one’s personal thoughts on quitting smoking with a machine that responds empathically to one’s input may create an “uncanny” feeling [58] because the program acts like a human without being one. Even though reports from users of *Endre* so far indicate to the contrary that they respond positively to the “mixture” of Man and machine, this is an area that will require further research.

### Future Directions

*Endre* and the theoretical model presented here will be evaluated in forthcoming studies. Because the application of the model to the program is made explicit, it is possible to test. Empirical investigations may in turn influence or alter the theoretical model or its recommended application to a program.

In one current Randomized Controlled Trial (RCT), the lapse management component will be evaluated by randomly allocating participants to one version of the program with the lapse management component and one version without it. The results of this RCT will tell us whether providing immediate help to users who have had a lapse can significantly improve their success rate. We also plan to collect indicators on working alliance and on internal motivation.

Another ongoing project is a qualitative study on the users’ working alliance to *Endre*. The goal of this study is to explore the nature of the working alliance because it is not given that working alliance to a fully automated program is identical to the working alliance to a human therapist. It is only when we can be convinced of the nature of working alliance to a fully automated program that it will be truly meaningful to test its importance for eHealth-assisted behavior change.

Finally, although we have argued that *Endre* simulates support of a working alliance, internalized motivation, and lapse preparation and lapse management, we do not know to what extent this simulation is successful for the user. One might establish simulation success through RCTs as the one described previously and compare the results with comparable findings from the counseling literature.

### Conclusions

We have demonstrated how *Endre*, a fully automated eHealth program, through interactivity and individual tailoring emulate 3 effective mechanisms of face-to-face counseling. By having used intervention mapping to systematically break down *Endre* into smaller components and showed how the program simulates a counselor’s support of a working alliance, internalized motivation, and lapse preparation and lapse management, our analysis is an example of how knowledge of what works in eHealth programs may be deepened by interpreting them in light of therapeutic processes. We suggest that the combination of these 3 therapeutic processes may result in a synergistic effect. Based on the analysis, we believe the combined support of a working alliance, internalization of motivation, and lapse preparation and management should be an element in the “black box” of automated eHealth behavior change programs that will make them more effective.

## Multimedia Appendix 1

Overview of the themes in Endre.

## Multimedia Appendix 2

The postquit day lapse management component of Endre.

## Multimedia Appendix 3

Change model from the development of Endre.

## Multimedia Appendix 4

Computerized motivational interviewing in Endre.

## Abbreviations

CMI: computerized motivational interviewing
MI: motivational interviewing
RCT: randomized controlled trial
SMS: short message service
