# Supplementary material for: How a Fully Automated eHealth Program Simulates Three Therapeutic Processes: A Case Study
Source: J Med Internet Res. 2016 Jun 28;18(6):e176. doi: 10.2196/jmir.5415 (PMC4942686; doi:10.2196/jmir.5415)
Supplement: Supplementary file 1 [file jmir_v18i6e176_app1.pdf]

Overview of the sessions in Endre

Session number	Topic	Content
**PREPARATION PHASE**		
**1**	Introduction	Introduction of the program.<br>The user may choose a special theme that will be addressed during the program:<br>1. Managing one's weight as smoke-free<br>2. Handling stress as smoke-free<br>3. Handling strong emotions as smoke-free<br>4. Establishing a smoke-free identity<br>5. Being smoke-free/smoking at parties.
**2**	Eliciting change-talk:<br>Reasons and need	• Elicits and reflects the user's reasons for quitting.<br>• Asks the user how important quitting is to her ("need ruler" – see "confidence ruler" in Multimedia Appendix 3).
**3**	Handling smoking urges	• Strategies for handling the urge to smoke.<br>• The user gets general advice and is asked to make personalized "smoking urge strategies".<br>• The user also gets a «home-assignment»: To pay attention to and note down her «smoking cues».
**4**	Making a quit plan<br>Eliciting change-talk:<br>Commitment	Making a plan for the quit attempt.<br>One of two possibilities:<br>1. If the user has a clear plan, she is asked to write it down.<br>2. If the user wants help in making a plan, she is guided through five questions:<br>a) "Will you reduce your cigarette smoking before quitting, or will you quit «cold turkey»?"<br>b) «What do you need to do the day before you quit?»<br>c) «What will you do with any cigarettes you might have left?»<br>d) «What will you do the day you quit?»<br>e) «How will you make sure to take care of yourself along the way?»<br>f) «What can you do when you get the urge to smoke?»<br>g) "Endre" adds how the user plans to handle smoking urges (user input from the last session).<br>After the plan is made, the user is asked whether or not she intends to follow through with it ("commitment ruler" – see "confidence ruler" in

		Multimedia Appendix 3).
**5**	Social support and public commitment	• Choosing a «support person» from the user's social network.<br>• Telling others about quitting (making a public commitment).<br>• If the user does not have anyone to ask for a "support person", "Endre" says that she is fully capable of quitting on her own, together with "his" support.
**6**	«Smoking cues» and withdrawal symptoms	• Following up on the «home-assignment»: What are your «smoking cues»?<br>• What withdrawal symptoms to expect, and how to minimize them.
**7**	Values and goals exploration	Identifying the user's life values. Asking the user how smoking and quitting smoking suit these values.
**8**	Psycho-education: Lapse management	The difference between a lapse and a relapse.<br>How to go back to quitting if you experience a lapse.
**9**	High-risk situations	• Identifying high-risk situations, taking the user's «smoking cues» as a starting point.<br>• Making a plan for handling high-risk situations. The user gets general advice (avoid the situation, leave the situation or use a "smoking urge strategy") and is asked to make a personalized plan.<br>• Confidence ruler: Staying smoke-free in high-risk situations.
**10**	Eliciting change-talk: Self-efficacy	Eliciting and strengthening the user's belief, or self-efficacy, in her ability to quit smoking («confidence ruler»).
**QUITTING PHASE**		
**11**	Confirm quit-attempt	The user is asked if she has quit smoking.<br>1. If the user answers yes, "Endre" congratulates and asks about her self-efficacy for staying smoke-free the rest of the day. The user then moves on to the follow-up phase.<br>2. If the user answers no, she is reminded of her plan to quit (user input from earlier session).
**12**	Confirm quit-attempt	If the user answered no last session, she receives a new session the next day asking if she has quit.<br>1. If the user answers yes, "Endre" congratulates and asks about her self-efficacy for staying smoke-free the rest of the day. The user then moves on to the follow-up phase.

		2. If the user answers no, “Endre” asks if she intends to quit tomorrow.<br>a) If the user answers yes, the session is ended.<br>b) If the user answers no, “Endre” asks if this is because tomorrow for some reason is not a good day, or if the user is feeling unsure about quitting.<br>1. If the user answers that tomorrow is not a good day, “Endre” accepts, but says that “he” will still ask her if she has quit tomorrow, just in case.<br>2. If the user answers that she is unsure about quitting, “Endre” offers help (self-efficacy or motivation).
**13**	Confirm quit-attempt	If the user answered that she had not quit last session, she receives a new session the next day asking if she has quit.<br>1. If the user answers yes, “Endre” congratulates and asks about her self-efficacy for staying smoke-free the rest of the day. The user then moves on to the follow-up phase.<br>2. If the user answers no, “Endre” asks her if this is because she wants to quit, but does not feel ready yet; or if she is unsure whether or not she wants to quit.<br>a) If the user answers that she does not feel ready, “Endre” offers advice. The advice is to stop postponing, because the time for quitting may never be “right”.<br>b) If the user answers that she is unsure whether or not she wants to quit, “Endre” offers help (self-efficacy or motivation).
**14**	Confirm quit-attempt	If the user answered that she had not quit last session, she receives a new session the next day asking if she has quit.<br>1. If the user answers yes, “Endre” congratulates and asks about her self-efficacy for staying smoke-free the rest of the day. The user then moves on to the follow-up phase.<br>2. If the user answers no, “Endre” says that

		"he" believes in her, and that "he" will ask again tomorrow. The session is repeated until the user confirms having quit (or stops logging on).
**FOLLOW-UP PHASE**		
**15**	Eliciting change-talk: Self-efficacy	Confidence ruler: Self-efficacy for staying smoke-free for the rest of the day.
**16**	Eliciting change-talk: Self-efficacy	Confidence ruler: Self-efficacy for staying smoke-free for the rest of the day.
**17**	Special theme	The user's special theme, chosen in session one. One of the following:<br>• Weight: Making a plan for maintaining one's weight when quitting smoking.<br>• Stress: Learning coping skills for handling stress without smoking.<br>• Strong emotions: Learning coping skills for handling strong emotions without smoking.<br>• Identity: Identity exercise: Who am I as smoke-free?<br>• Smoking at parties: Staying smoke-free (or not) at parties, and how.
**18**	High-risk situations	The user is asked if she has been in any high-risk situations. If she has, she is asked if she has been smoke-free in any of them.
**19**	Detecting and stopping a lapse	How to stop a lapse before it occurs.<br>Reminder: The user's reasons for quitting.
**20**	Social support	How to get the best support from the "support person". The user is asked to specify what she needs (both of practical help and emotional support), and to specify if there is anything she wants the "support person" to *stop* doing. She is encouraged to share these thoughts with her "support person".<br><br>Users who did not find a "support person" get a different session, where "Endre" acts as a support person: Reminding her to use the program for support and inspiration, and telling her how well she has done so far on her quit attempt.
**21**	Internal attribution of success	Reminder: Everything the user has achieved is due to her own efforts.
**22**	Eliciting change-talk: Self-efficacy	Confidence ruler: Self-efficacy for staying smoke-free.
**23**	Special theme	A new session on the user's special theme.

		The user gets one of the following:<br><br>• Weight: Opportunity to reconsider one's plan for not gaining weight.<br>• Stress: Asks for the user's coping strategies for handling stress.<br>• Strong emotions: Asks for the user's coping strategies for handling strong emotions.<br>• Identity: Asks if there are any situations in which the user feels smoke-free.<br>• Smoking at parties: Follows up on the user's input from the first session (whether or not she planned to stay smoke-free at parties).<br><br>All users are also asked whether parties might be a high-risk situation for lapsing.
**24**	Reminder: Reasons	"Endre" reminds the user of her reason for quitting. The user may write a new most important reason if she wants. The user may also write a new text on how quitting smoking relates to her values.
**25**	High-risk situations	The user is asked if she has been in any high-risk situations. If necessary, the plan for handling high-risk situations is revised.<br>Confidence ruler: Staying smoke-free in high-risk situations.
**26**	Eliciting change-talk: Self-efficacy	Confidence ruler: Self-efficacy for being smoke-free two years from now.
**27**	Internal attribution of success	Reminder: Everything the user has achieved is due to her own efforts.
**28**	Final session	Sums up the lessons from the program and wishes the user good luck.
