# Supplementary material for: How a Fully Automated eHealth Program Simulates Three Therapeutic Processes: A Case Study
Source: J Med Internet Res. 2016 Jun 28;18(6):e176. doi: 10.2196/jmir.5415 (PMC4942686; doi:10.2196/jmir.5415)
Supplement: Supplementary file 2 [file jmir_v18i6e176_app2.pdf]

Lapse management: SMS messages and web-session

After quitting day, the user's smoking status is monitored by an automatically generated "Checking in"-SMS from "Endre" every evening. The user is requested to answer the "Checking in"-SMS, replying NO if she has had a cigarette and YES if she has been entirely smoke-free. If the user answers YES (no lapse), she immediately receives a "Well done"-SMS with an affirmation. If the user answers NO (reports a lapse) she receives a "First aid"-SMS intended to reduce the negative emotions and catastrophic cognitions of the abstinence violation effect (AVE) [1] through accurate empathy [2] and underscoring the importance of the user's choice. The "First aid"-SMS contains a link to the web-based lapse-management session. If the user for some reason cannot use the link (e.g. she does not have a smartphone), she gets the lapse management session the next time she logs on to the program.

The web-based lapse-management session is tailored to the user's input through small intermediate mini sessions, or snippets. The first snippet is quite short, only meant to clarify whether the user needs further help. If help is needed, the user may choose between four alternatives. The user's choice determines the build-up of the remaining session. An overview of the seven snippets' content can be found in the table to the right. The flow-chart of the session can be viewed beneath.

SMS-type	Example	Number of versions
"Checking-in"	"Hi, [user's name]! How has your day been? Have you been smoke-free? If yes, please answer YES. If you've had a slip-up, please answer NO. Best, Endre."	28
"Well-done"	"Wonderful! I hope you're proud of yourself! Best, Endre."	28
"First-aid"	"Thank you for being honest! A slip-up doesn't have to mean that much. You can still choose to be smoke-free, if that's what you want. Click on the link in this SMS, or log on to the program. It's easier to talk about this on the webpage. (link)"	10

Snippet	Description
**Reattribution [1]**	Mandatory for all users. Involves attributing the lapse to the situation [1] – where the user was, who she was with, what she did, what she felt.
**Abstinence Violation Effect [1]**	Only provided if the user identifies with a description of the Abstinence Violation Effect (see flow-chart). Involves:<br>- Empathic reflection [2]<br>- Thinking about past mastery experiences [2]<br>- Normalization<br>- Replacing counter-productive thoughts [3]
**Exploring ambivalence [2]**	Only provided if the user indicates that she is unsure whether she wants to keep quitting. Explores negative and positive aspects of quitting [2].
**Choose**	Mandatory. The user is asked, on the basis of what has been "discussed", to decide whether to keep quitting or keep smoking. If the user has not explored ambivalence, "Unsure" is an option. If she already has explored ambivalence, she may only answer "Keep smoking" or "Keep quitting".
**Act**	Mandatory (if the user chooses to keep quitting). The user is encouraged to get rid of remaining cigarettes, asked if she is in a high-risk situation, and guided back to a safe situation if necessary.
**Re-planning [2] and asking about social support**	Mandatory (if the user chooses to keep quitting). The user is shown her input from the reattribution-snippet (above) and is asked to make a plan [2] on how to avoid a lapse in a similar situation in the future. The user is also asked if she finds it difficult to explain the lapse to significant others, and is offered advice on how to do so if needed.
**Self-efficacy [2]**	Mandatory (if the user chooses to keep quitting). Confidence-ruler [2] asking about self-efficacy for staying smoke-free.

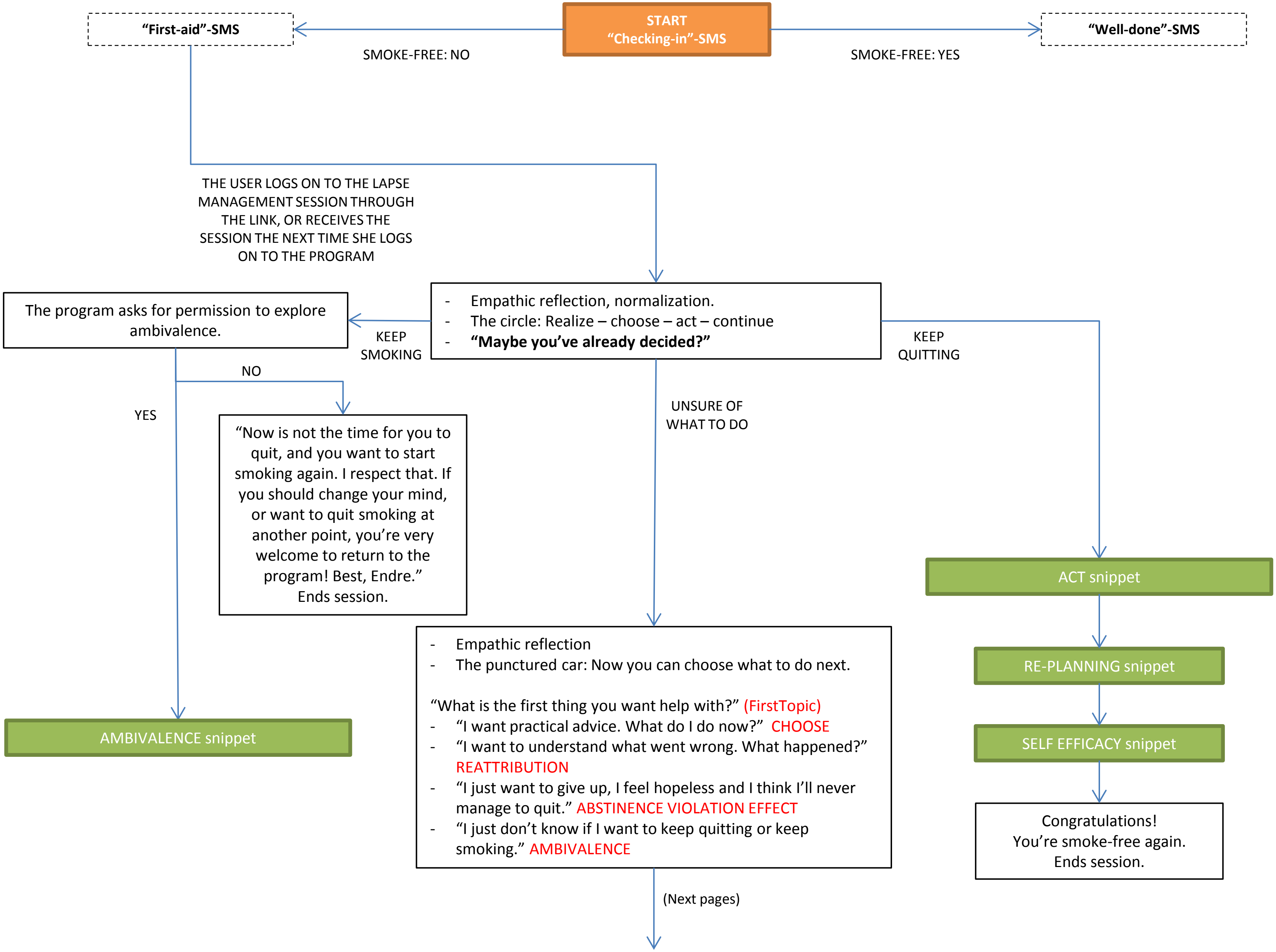

START
"Checking-in"-SMS
"First-aid"-SMS
SMOKE-FREE: NO
"Well-done"-SMS
SMOKE-FREE: YES
THE USER LOGS ON TO THE LAPSE MANAGEMENT SESSION THROUGH THE LINK, OR RECEIVES THE SESSION THE NEXT TIME SHE LOGS ON TO THE PROGRAM
- Empathic reflection, normalization.
- The circle: Realize – choose – act – continue
- "Maybe you've already decided?"
KEEP SMOKING
The program asks for permission to explore ambivalence.
NO
YES
"Now is not the time for you to quit, and you want to start smoking again. I respect that. If you should change your mind, or want to quit smoking at another point, you're very welcome to return to the program! Best, Endre." Ends session.
AMBIVALENCE snippet
UNSURE OF WHAT TO DO
- Empathic reflection
- The punctured car: Now you can choose what to do next.
"What is the first thing you want help with?" (FirstTopic)
- "I want practical advice. What do I do now?" CHOOSE
- "I want to understand what went wrong. What happened?" REATTRIBUTION
- "I just want to give up, I feel hopeless and I think I'll never manage to quit." ABSTINENCE VIOLATION EFFECT
- "I just don't know if I want to keep quitting or keep smoking." AMBIVALENCE
(Next pages)
KEEP QUITTING
ACT snippet
RE-PLANNING snippet
SELF EFFICACY snippet
Congratulations! You're smoke-free again. Ends session.

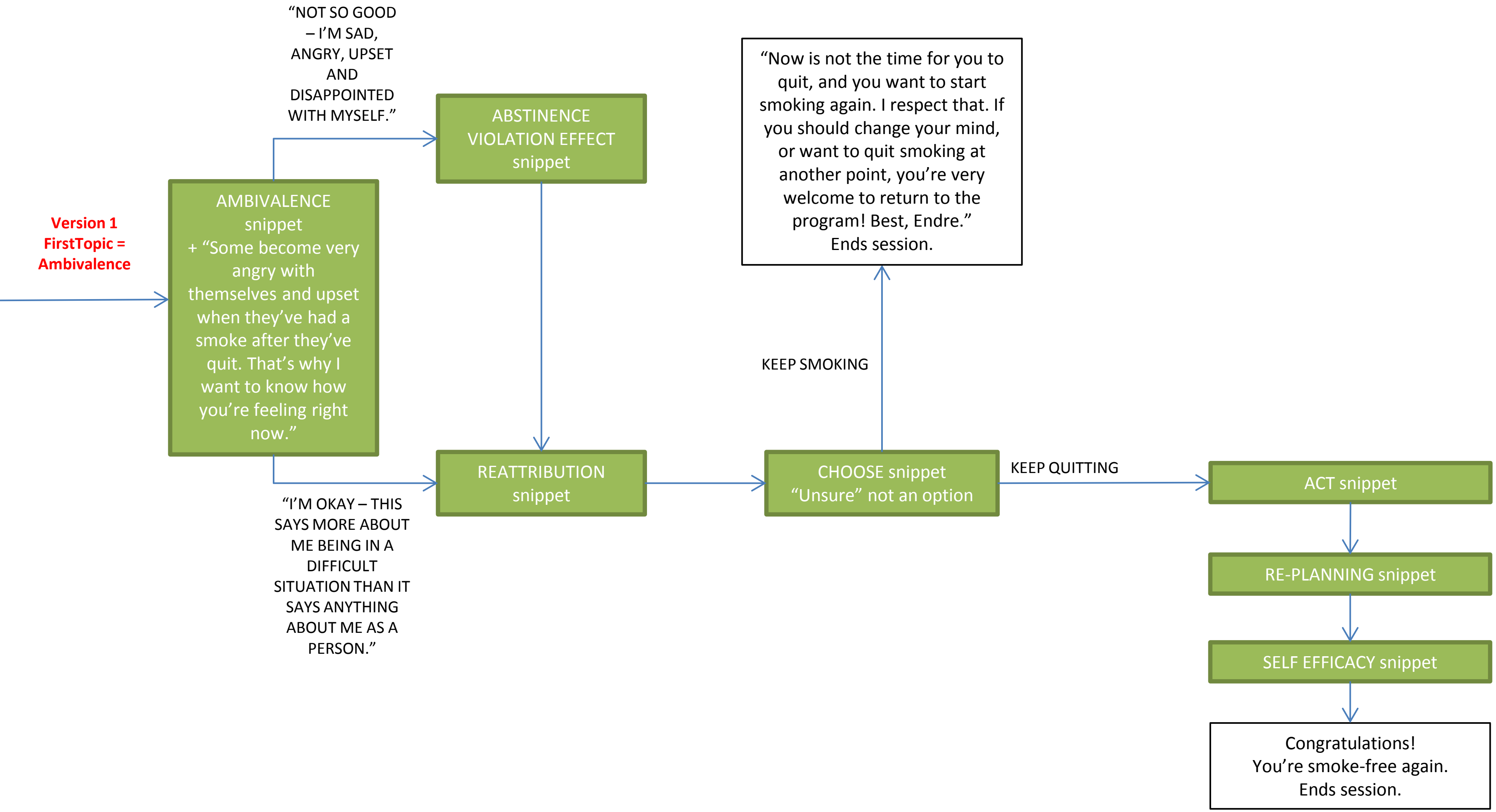
Version 1
FirstTopic =
Ambivalence
AMBIVALENCE snippet + "Some become very angry with themselves and upset when they've had a smoke after they've quit. That's why I want to know how you're feeling right now."
"NOT SO GOOD – I'M SAD, ANGRY, UPSET AND DISAPPOINTED WITH MYSELF."
ABSTINENCE VIOLATION EFFECT snippet
"I'M OKAY – THIS SAYS MORE ABOUT ME BEING IN A DIFFICULT SITUATION THAN IT SAYS ANYTHING ABOUT ME AS A PERSON."
REATTRIBUTION snippet
CHOOSE snippet "Unsure" not an option
KEEP SMOKING
"Now is not the time for you to quit, and you want to start smoking again. I respect that. If you should change your mind, or want to quit smoking at another point, you're very welcome to return to the program! Best, Endre." Ends session.
KEEP QUITTING
ACT snippet
RE-PLANNING snippet
SELF EFFICACY snippet
Congratulations! You're smoke-free again. Ends session.

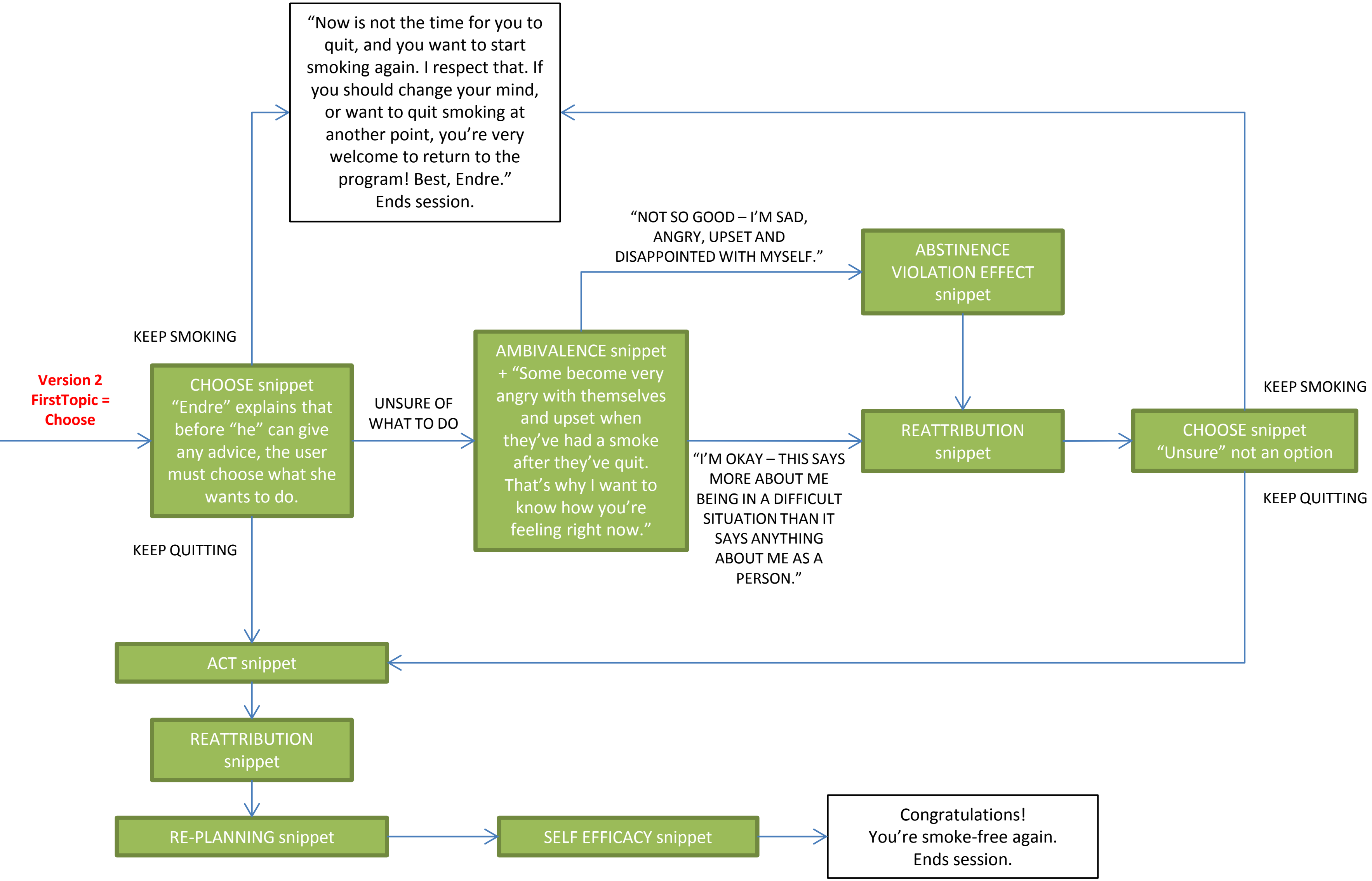

Version 2 FirstTopic = Choose
CHOOSE snippet “Endre” explains that before “he” can give any advice, the user must choose what she wants to do.
KEEP SMOKING
“Now is not the time for you to quit, and you want to start smoking again. I respect that. If you should change your mind, or want to quit smoking at another point, you’re very welcome to return to the program! Best, Endre.” Ends session.
UNSURE OF WHAT TO DO
AMBIVALENCE snippet + “Some become very angry with themselves and upset when they’ve had a smoke after they’ve quit. That’s why I want to know how you’re feeling right now.”
“NOT SO GOOD – I’M SAD, ANGRY, UPSET AND DISAPPOINTED WITH MYSELF.”
ABSTINENCE VIOLATION EFFECT snippet
“I’M OKAY – THIS SAYS MORE ABOUT ME BEING IN A DIFFICULT SITUATION THAN IT SAYS ANYTHING ABOUT ME AS A PERSON.”
REATTRIBUTION snippet
CHOOSE snippet “Unsure” not an option
KEEP SMOKING
KEEP QUITTING
KEEP QUITTING
ACT snippet
REATTRIBUTION snippet
RE-PLANNING snippet
SELF EFFICACY snippet
Congratulations! You’re smoke-free again. Ends session.

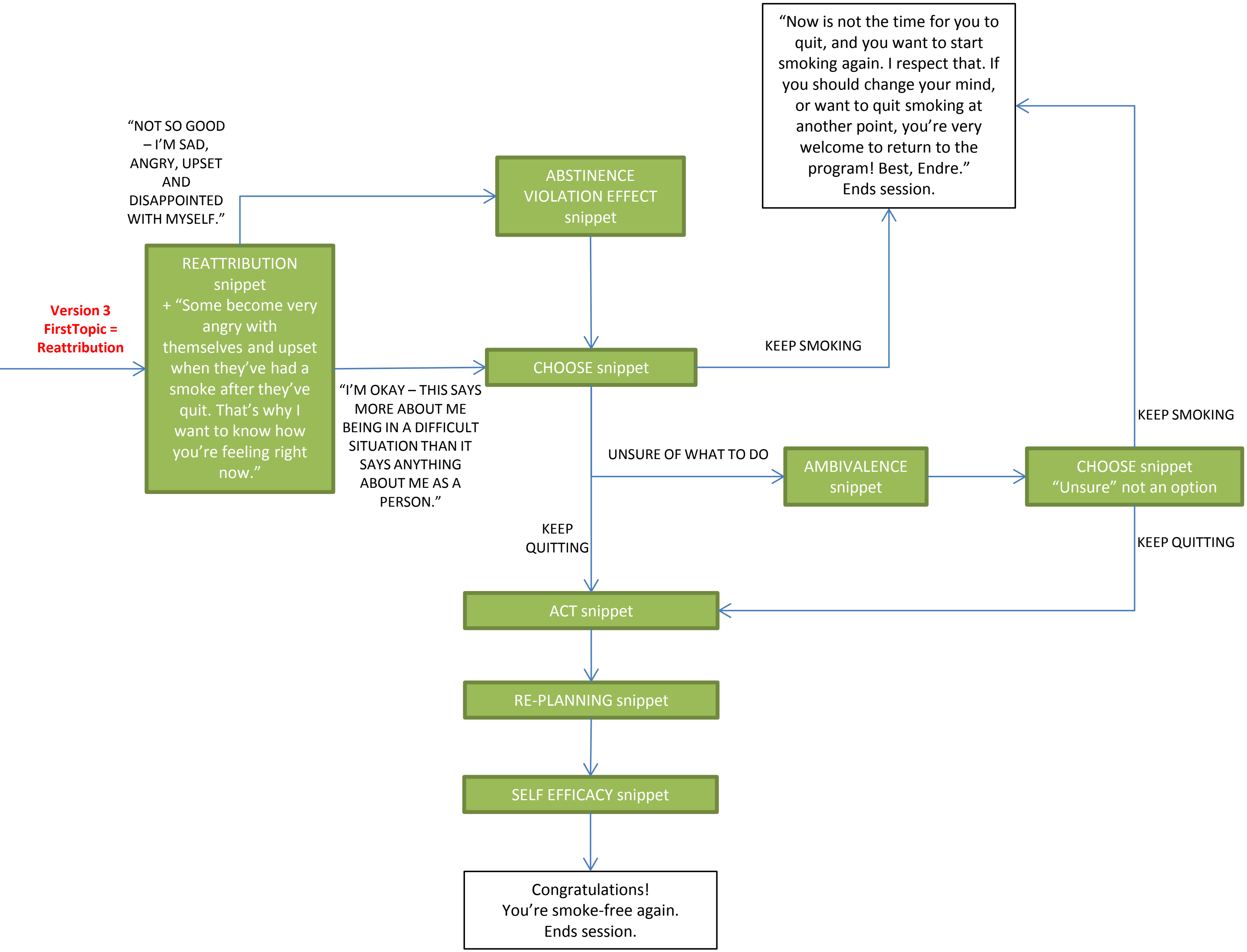

Version 3
FirstTopic =
Reattribution
REATTRIBUTION snippet + "Some become very angry with themselves and upset when they've had a smoke after they've quit. That's why I want to know how you're feeling right now."
"NOT SO GOOD – I'M SAD, ANGRY, UPSET AND DISAPPOINTED WITH MYSELF."
ABSTINENCE VIOLATION EFFECT snippet
"I'M OKAY – THIS SAYS MORE ABOUT ME BEING IN A DIFFICULT SITUATION THAN IT SAYS ANYTHING ABOUT ME AS A PERSON."
CHOOSE snippet
KEEP SMOKING
"Now is not the time for you to quit, and you want to start smoking again. I respect that. If you should change your mind, or want to quit smoking at another point, you're very welcome to return to the program! Best, Endre." Ends session.
UNSURE OF WHAT TO DO
AMBIVALENCE snippet
CHOOSE snippet "Unsure" not an option
KEEP SMOKING
KEEP QUITTING
KEEP QUITTING
ACT snippet
RE-PLANNING snippet
SELF EFFICACY snippet
Congratulations! You're smoke-free again. Ends session.

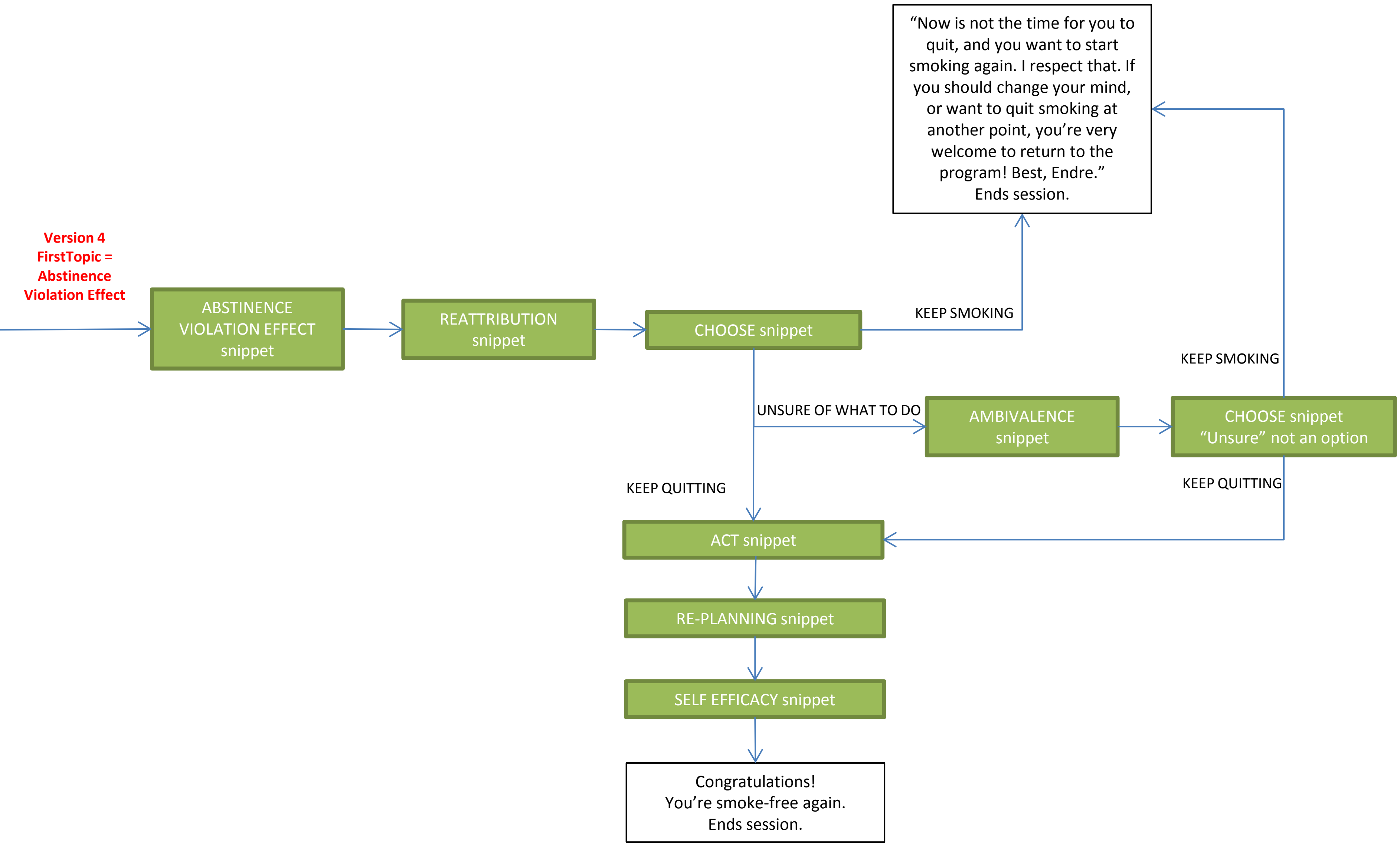
Version 4
FirstTopic =
Abstinence
Violation Effect
ABSTINENCE VIOLATION EFFECT snippet
REATTRIBUTION snippet
CHOOSE snippet
KEEP SMOKING
“Now is not the time for you to quit, and you want to start smoking again. I respect that. If you should change your mind, or want to quit smoking at another point, you’re very welcome to return to the program! Best, Endre.”
Ends session.
KEEP SMOKING
UNSURE OF WHAT TO DO
AMBIVALENCE snippet
CHOOSE snippet
“Unsure” not an option
KEEP QUITTING
KEEP QUITTING
ACT snippet
RE-PLANNING snippet
SELF EFFICACY snippet
Congratulations!
You’re smoke-free again.
Ends session.

References

[1] Marlatt G, Donovan D, editors. Relapse Prevention: Maintenance Strategies in the Treatment of Addictive Behaviors. Second edi. New York: The Guilford Press; 1985.

[2] Miller WR, Rollnick S. Motivational interviewing: Helping people change. Third edit. New York: Guilford Press; 2012.

[3] Beck AT, Haigh EAP. Advances in Cognitive Theory and Therapy: The Generic Cognitive Model*. Annu Rev Clin Psychol 2014;10(1):1–24.
