# Supplementary material for: How a Fully Automated eHealth Program Simulates Three Therapeutic Processes: A Case Study
Source: J Med Internet Res. 2016 Jun 28;18(6):e176. doi: 10.2196/jmir.5415 (PMC4942686; doi:10.2196/jmir.5415)
Supplement: Supplementary file 3 [file jmir_v18i6e176_app3.pdf]

Original change model of "Endre"

Health behaviour: Quitting smoking and staying smoke-free through the help of "Endre"

PERFORMANCE OBJECTIVES	PERSONAL DETERMINANTS				
	Internalized motivation				
	Relatedness		Competence		Autonomy (Aut)
	To the program (RelP)	To social network (RelSN)	Skills (Ski)	Self-efficacy (SE)	
**PO1: Decide to quit smoking and plan how to do it.**	**RelP.1a:** Experience the program as a social actor.<br><br>**RelP.1:** Experience the program as:<br>b) Accessible.<br>c) Potentially helpful.<br>d) Empathic, warm, genuine, and unconditionally accepting.<br>e) Having general expertise on smoking	**RelSN.1a:** Make a public commitment to the quit attempt.<br><br>**RelSN.1b:** Choose a "support person" from one's personal network.	**Ski.1a:** Identify personal smoking cues and be able to detect smoking urges and cravings early.<br><br>**Ski.1b:** Prepare to handle cravings.<br><br>**Ski.1c:** Make an action and coping-plan for the quit attempt.	**SE.1a:** Believe it to be possible to quit smoking and stay smoke-free.<br><br>**SE.1b:** Express confidence in one's ability to execute the cessation plan.	**Aut.1a:** Commit to the quit attempt.<br><br>**Aut.1b:** Decide whether or not to make a public commitment to the quit attempt.<br><br>**Aut.1c:** Decide whether or not to engage a "support person" in the cessation attempt.<br><br>**Aut.1d:** Choose how to make the quitting plan (by oneself or a more guided version).

	cessation that can be combined with the user's expertise on herself.<br>f) Trustworthy.<br>g) Responsive to user input.<br><br>**RelP.2h:**<br>Choose a special theme to influence the program content.<br><br>**RelP.2i:** Understand how to use the program and do the exercises.		**Ski.1d:** Identify one's high-risk situations, and make an action and coping-plan for handling them.		**Aut.1e:** Combine the advice of the program with one's own style and preferences.
**PO2: Initiate the quit attempt and stay smoke-free for the first three days.**	**RelP.1d-g, i**	**RelSN.2:** Request support from the "support person" as needed.	**Ski.2a:** Implement action- and coping-plan for the quit attempt.<br><br>**Ski.2b:** Get rid of remaining cigarettes and smoking accessories.	**SE.2:** Express confidence in one's ability to stay smoke-free the first three days.	**Aut.2a:** Revise the action- and coping-plan if needed.<br><br>**Aut.2b:** Decide whether or not to get rid of remaining cigarettes, or whether to make the cigarettes less accessible.

			**Ski.2c:** Withstand cravings and cope with withdrawal symptoms.		
**PO3: Maintain the quit attempt over time (from day 4 and onwards).**	**RelP.3:** Experience the program as:<br>a) Sensitive and adjustable if new needs emerge.<br>b) Suiting one's own preferences and style.<br><br>**RelP.3c:** Continue with the program for as long as needed, and if necessary return to the program after a period of disengagement.	**RelSN.3:** Ask the "support person" for help and support/reinforcement to the extent that is needed.	**Ski.3a:** Identify and counteract thought-patterns that could lead to a (re)lapse ("lapse signatures").<br><br>**Ski.3b:** Follow plans for high-risk situations.<br><br>**Ski.3c:** Stay smoke-free, also in social situations and at parties.<br><br>**Ski.3d:** Be able to imagine one-self as smoke-free.	**SE.3a:** Express confidence in one's ability to stay smoke-free in high-risk situations.<br><br>**SE.3b:** Express confidence in staying smoke-free in the long run.	**Aut.3a:** Decide to what degree, when and how the "support person" is needed.<br><br>**Aut.3b:** Know that staying smoke-free or not is one's own choice.<br><br>**Aut.3c:** Attribute success in the cessation attempt internally.<br><br>**Aut.3d:** Decide not to think too far ahead if doing so creates counterproductive, negative stress.

**PO4: Resume the quit attempt after a lapse and use the lapse as a learning experience.**	**RelP.1d, f-g, RelP.3a-b**	**RelSN.4:** Be able to explain the difference between a lapse and a relapse to significant others in order to gain their understanding for the lapse and support for the continued quit attempt.	**Ski.4a:** Know the difference between a lapse and a relapse.<br><br>**Ski.4b:** Get rid of any spare cigarettes after a lapse.<br><br>**Ski.4c:** Resist new urges to smoke.	**SE.4:** Express confidence in one's ability to continue with the quit attempt after a lapse.	**Aut.4a:** Know that whether to keep smoking or keep quitting is a matter of one's own choice.<br><br>**Aut.4b:** Know that whether or not to be completely abstinent of cigarettes is one's own choice.

Comments

All the change objectives under the determinant “Relatedness to the program”, except RelP.1a, are derived from Barazzone and colleagues’ paper on working alliance in fully automated computerized Cognitive Behavioural Therapy programs [1]. Change objectives SE.1a and Aut.1e is also based on this study. RelP.1a is derived from the work of Bickmore and colleauges [2].

Change objective RelSN.3 is based on a Cochrane review [3] that suggests two types of partner behaviours as important: Helping behaviour (“such as talking the smoker out of taking a cigarette”) and emotional reinforcement of the individual’s efforts [3].

Change objective Ski.3a is greatly inspired by a Norwegian book on Motivational Interviewing by Barth and colleagues [4], as well as a book on mindfulness [5].

References

1. Barazzone N, Cavanagh K, Richards D a. Computerized cognitive behavioural therapy and the therapeutic alliance: a qualitative enquiry. Br J Clin Psychol [Internet] 2012 Nov [cited 2013 Sep 16];51(4):396–417. Available from: http://www.ncbi.nlm.nih.gov/pubmed/23078210 PMID: 23078210

2. Bickmore T, Gruber A, Picard R. Establishing the computer–patient working alliance in automated health behavior change interventions. Patient Educ Couns [Internet] 2005;59(1):21–30. Available from: http://linkinghub.elsevier.com/retrieve/pii/S0738399104003076

3. Park E, Tudiver F, Campbell T. Enhancing partner support to improve smoking cessation (Review). Cochrane database Syst Rev 2012;(7).

4. Barth T, Børtveit T, Prescott P. Endringsfokusert rådgivning. Gyldendal akademisk; 2003.

5. Kabat-Zinn J. Wherever you go, there you are: Mindfulness meditation in everyday life. Hyperion, editor. 1994.
