# Supplementary material for: How a Fully Automated eHealth Program Simulates Three Therapeutic Processes: A Case Study
Source: J Med Internet Res. 2016 Jun 28;18(6):e176. doi: 10.2196/jmir.5415 (PMC4942686; doi:10.2196/jmir.5415)
Supplement: Supplementary file 4 [file jmir_v18i6e176_app4.pdf]

Computerized Motivational Interviewing (cMI) in "Endre"

<table>
<tr><th>MI-technique</th><th>cMI-application</th></tr>
<tr><td>The "spirit" of MI [1]</td><td>The "spirit" of MI consists of collaboration, acceptance (absolute worth, autonomy, accurate empathy, and affirmation), evocation, and compassion [1].

Collaboration and autonomy is communicated throughout the program: Whenever "Endre" gives an advice, it is underscored that ultimately the user knows what is best for her and most make her own decisions.
Absolute worth: In the very first session, "Endre" reveals that although "he" "pretends to be" a real person, "he" in fact is not, and "he" would not want to deceive the user to believe otherwise. This communicates that the user is of absolute worth and should not be led under false pretence.
Accurate empathy:

"Endre" communicates with accurate empathy – within the restriction that "he" can only reflect with accurate empathy when the user is given pre-defined multiple choice alternatives (see below).
Accurate empathy is also communicated through the multiple choice alternatives themselves. For example, when "Endre" asks the user how certain she is of staying smoke-free the first couple of days (see below), the statements representing the five degrees of self-efficacy reflects an empathic understanding of a large range of emotional states (e.g. "It sounds terribly difficult, but I will do my best").

Affirmations: "Endre" provides the user with affirmations (genuine comments on the user's strengths and accomplishments), e.g. "Congratulations, you've done quite a bit of work today towards your aim of becoming smoke-free!", or "So you've tried quitting before – that means you've got some experience to draw from now". Two sessions are entirely devoted to an affirmation: Underscoring that the user's accomplishments are due to own efforts, and nobody else's.
Evocation: The entire program is based on asking the user to give her opinions, her reasons, and her plans.
Compassion: Compassion is perhaps communicated best through the fact that the first author has written all the text for the program, drawing upon her experience with MI-based telephone-counselling. The compassion is not mechanically put into different parts of the program, but is conveyed through the actual compassion for the prospective user.
The spirit of MI is also sought communicated through the program's visual design and interface.
</td></tr>
</table>

**Open questions, reflections and summaries [1]**	"Endre" asks questions, and the user may sometimes answer through a text box, sometimes through multiple choice alternatives. Multiple choice questions allow for *complex reflections* of the user's answer, because the answer is "understood" by the program [2] (this includes communicating with accurate empathy, as described above). Freely written text in a text box, on the other hand, gives the user the opportunity to compose a personal answer. These answers, however, can only be *directly reflected* back to the user, without taking into account their actual contents [2]. Friederichs and colleagues recommend combining multiple choice questions and open questions with text boxes for freely written text [2]. In "Endre", questions are sometimes phrased with text box answers, sometimes with multiple-choice, but they are rarely combined. The reason for this is because the mere number of questions in the program would make the "double" questions tedious for the user, and potentially disturb the flow of the "conversation".
**Eliciting and reflecting change-talk**	"Endre" elicits and reflects change-talk, or "language that signals movement towards change" [1]. There are different types of change-talk: Desires, ability, reasons, need, commitment, activating language, and taking small steps [1]. Ability, desires/reasons, need, commitment, and taking small steps are all elicited in "Endre" – for details, see below.<br><br>"Endre" reflects the user's change-talk; directly, if the change-talk is in a text box, more complex if the change-talk is expressed through multiple-choice alternatives.
**Eliciting change-talk: Ability/self-efficacy ("Confidence ruler"), need, and commitment [1]**	The user is asked to indicate her self-efficacy in achieving a specific goal by choosing a statement representing five degrees of self-efficacy ranging from very low to very high. After having chosen a statement, the user is asked to type in why she did not choose a statement reflecting lower self-efficacy. This reason is subsequently reflected back to her. By asking the user to justify her choice, reasons that speak towards changing are elicited [1]. If the user chooses the lowest degree of confidence, she is either asked what she can do to feel more confident – also eliciting change-talk [1], or she is offered help.<br>At some occasions, the user may choose a number from 1-5 instead of choosing between statements [3]. One benefit of using statements instead of numbers is that "Endre" by that defines what is meant by the lowest degree of self-efficacy, making it less likely that the user will choose that alternative. In most cases, the lowest alternative represents an extreme that most people will not identify with (i.e. "It's absolutely hopeless and I've already given up"). If the user reads this alternative and does not choose it because she finds it too extreme, she is indirectly strengthening her change-talk. This might be seen as a version of "agreeing with a twist", a strategy where the therapist agrees with the client's sustain-talk as a way of making her see it in a different light [1].<br><br>Change-talk concerning need, or how important quitting is to the user, as well as commitment to the quit plan, is elicited through a "ruler" in the same way as change-talk concerning ability.

**Eliciting change talk: Commitment and taking steps [1]**	When a user agrees to follow "Endre's" (general) advice, she is sometimes asked to specify how she will do it. For example, if the user agrees to get rid of any remaining cigarettes when she has quit, "Endre" asks what she will do with them. When the user specifies how she will do an action leading towards quitting, such as getting rid of remaining cigarettes, she is strengthening her commitment to doing it, and this is therefore a form of change-talk.
**Eliciting change-talk: Reasons [1]**	The user is asked about her reasons for quitting smoking. "Endre" asks the user to write down her most important reason for quitting, starting the sentence with "I want to stop smoking because…" By completing the sentence with her personal reason, the user is producing change-talk.<br><br>The user is also asked to choose more reasons from a list of alternatives, and "Endre" reflects these reasons back to her, elaborating on them (complex reflection).
**Handling sustain-talk or discord [1]**	"Endre" allows the expression of sustain-talk (reasons for upholding the status quo) or discord (dissatisfaction with therapy) as multiple-choice alternatives where sustain-talk or discord could be expected as a likely response to "Endre's" question. If the user chooses a sustain-talk/discord alternative, "Endre" provides a complex, empathically accurate reflection. Depending on the context, "Endre" may normalize, offer help, follow up with more questions, or simply reflect and refocus [1]. For instance, if the user has expressed a low degree of self-efficacy, "Endre" offers to help through reminding the user of her reasons for quitting or how she might handle smoking urges. The user then may choose "Please remind me of my reasons for quitting", "Please remind me of how I can handle the cravings", or "None of these alternatives seem very helpful". The complex, empathic reflection provided by "Endre" is crafted to accurately reflect the response the user has given, but at the same time to communicate an appropriate amount of hope or normalization of the user's feelings [1].
**Asking for permission before giving advice/information [1]**	In MI, a counsellor should ask the client for permission before giving advice or information [1]; it should not be given out uninvited.<br>The cMI-equivalent of asking for permission is to require an action from the user before revealing the advice. This can be done by clicking on a video that gives the advice [3]. In "Endre", asking for permission is done in two different ways:<br><br>1. Multiple-choice alternatives. This is a close parallel to asking for permission in face-to-face conversations, requesting a "yes" or "no" from the client/user before continuing. For example, "Endre" may ask the user for permission before embarking on the topic of the day ("Today's session is going to be about revisiting your reasons for quitting. Is that okay for you?"). "Endre" may add that if the user says no, the session will be short because "he" has nothing else planned for today. The user may answer yes or no, and if she answers no, the session is ended.

	2. Hiding and showing text upon mouse click. The user is asked whether she wants an advice (or a piece of information). Clicking on the text reveals the advice or information, without leaving the page. If the user does not want to receive the advice or information, she can go to the next page without revealing the text.
**Exploring values and goals [1]**	Internalized motivation is characterized by the motivation being assimilated into the person's value- and belief-system [4]. In MI, one may use a "structured value exploration" as an exercise with the client [1]. This can also be done in a fully automated program [3]. In "Endre", a separate session is dedicated to exploring values and goals:<br>1. The user may choose from a list of values which values are important for her.<br>2. On the subsequent page, the user's choices are reflected back to her.<br>3. "Endre" asks the user to specify how (a) smoking and (b) quitting smoking relate to the values she has chosen. The user writes this down in a text box.<br>4. The user's text is reflected back to her.
**Exploring ambivalence [1]**	In MI, one should explore ambivalence only if the client is truly uncertain of what she wants [1]. For this reason, "Endre" only suggests exploring ambivalence if the user reports that she might want to start smoking again (expressed through multiple choice). When exploring ambivalence, "Endre":<br>1. Asks the user to describe positive aspects of smoking, or things that she (is going to) miss.<br>2. Asks the user to describe (personally) negative aspects of smoking.<br>3. Asks the user to describe negative aspects of quitting.<br>4. Asks the user to describe short term positive aspects of quitting.<br>5. Asks the user to describe long term positive aspects of quitting.<br>6. Summarizes the ambivalence exploration by showing all the text the user has typed in (direct reflection).<br>7. Asks the user to consider what she has written and decide whether she wants to continue quitting or start smoking again (multiple choice question, one answer possible).<br>8. If the user answers that she wants to keep quitting, "Endre" reacts with empathic gladness.<br>9. If the user indicates that she wants to start smoking again, "Endre" responds empathically and respects this.
**Developing a change plan [1]**	When helping the client to develop a change plan, Miller and Rollnick recommend that the therapist first establishes whether the client already has a clear plan, if she has an unclear plan, or if she has no plan at all. These different starting points call for different approaches by the therapist [1]. In "Endre", a separate session is dedicated to making a change plan. In this session, the user is first asked whether she already knows what to do (clear plan) or whether she would like some help in making a change plan (unclear plan / no plan at all).

	If the user indicates that she already has a plan, she is simply asked to write that plan down in a text box.<br><br>If the user indicates that she wants help in making the plan, she is guided through six different questions:<br>1. "Will you reduce your cigarette smoking before quitting, or will you quit «cold turkey»?"<br>2. «What do you need to do the day before you quit?»<br>3. «What will you do with any cigarettes you might have left?»<br>4. «What will you do the day you quit?»<br>5. «How will you make sure to take care of yourself along the way?»<br>6. «What can you do when you get the urge to smoke?»<br><br>Each question is followed by a text box, and the four fictional "quitters" provide "their" answers as examples.<br><br>Finally, "Endre" presents the user's answer to all six questions (direct reflection) as the user's cessation plan.

References

1. Miller WR, Rollnick S. Motivational interviewing: Helping people change. Third edit. New York: Guilford Press; 2012.

2. Friederichs S a H, Oenema A, Bolman C, Guyaux J, van Keulen HM, Lechner L. Motivational interviewing in a web-based physical activity intervention: questions and reflections. Health Promot Int [Internet] 2013 Oct 6 [cited 2014 May 28]; Available from: http://www.ncbi.nlm.nih.gov/pubmed/24101160 PMID: 24101160

3. Friederichs SA, Oenema A, Bolman C, Guyaux J, van Keulen HM, Lechner L. I Move: systematic development of a web-based computer tailored physical activity intervention, based on motivational interviewing and self-determination theory. BMC Public Health [Internet] 2014 Jan [cited 2014 Mar 23];14(1):212. Available from: http://www.pubmedcentral.nih.gov/articlerender.fcgi?artid=3944675&tool=pmcentrez&rendertype=abstract PMID: 24580802

4. Ryan RM, Deci EL. Self-determination theory and the facilitation of intrinsic motivation, social development, and well-being. Am Psychol [Internet] 2000;55(1):68–78. Available from: http://doi.apa.org/getdoi.cfm?doi=10.1037/0003-066X.55.1.68
